# Catch crop amendments and microbial inoculants differently modulate apple rhizosphere microbiomes and plant responses

**DOI:** 10.1093/femsec/fiaf055

**Published:** 2025-05-23

**Authors:** Kristin Hauschild, Adriana Giongo, Benye Liu, Doreen Babin, Elke Bloem, Ludger Beerhues, Traud Winkelmann, Kornelia Smalla

**Affiliations:** Institute for Epidemiology and Pathogen Diagnostics, Julius Kühn Institute (JKI) – Federal Research Centre for Cultivated Plants, 38104 Braunschweig, Germany; Institute for Epidemiology and Pathogen Diagnostics, Julius Kühn Institute (JKI) – Federal Research Centre for Cultivated Plants, 38104 Braunschweig, Germany; Leibniz Institute of Vegetable and Ornamental Crops (IGZ), 14979 Großbeeren, Germany; Institute of Pharmaceutical Biology, Technische Universität Braunschweig, 38016 Braunschweig, Germany; Institute for Epidemiology and Pathogen Diagnostics, Julius Kühn Institute (JKI) – Federal Research Centre for Cultivated Plants, 38104 Braunschweig, Germany; Institute for Crop and Soil Science, Julius Kühn Institute (JKI) – Federal Research Centre for Cultivated Plants, 38116 Braunschweig, Germany; Institute of Pharmaceutical Biology, Technische Universität Braunschweig, 38016 Braunschweig, Germany; Institute of Plant Genetics, Leibniz University Hannover, 30419 Hannover, Germany; Institute for Epidemiology and Pathogen Diagnostics, Julius Kühn Institute (JKI) – Federal Research Centre for Cultivated Plants, 38104 Braunschweig, Germany

**Keywords:** apple replant disease, greenhouse experiment, plant–soil feedback, root phytoalexins, root spatial gradient, *Tagetes patula*

## Abstract

Plant–soil feedback and soil microbial legacies play crucial roles in replanting success of apple. This study investigated how different soil amendment strategies influence these factors in replant disease-affected soil. Two approaches were tested: (i) the preculture and amendment of catch crops—either a single species, *Tagetes patula*, or a diverse catch crop mixture (CCM), and (ii) the inoculation of plant-beneficial microbes—bacteria, arbuscular mycorrhizal fungi, or their combination (SynC). Apple rootstock M.26 was grown for seven weeks in a greenhouse, and plant growth, soil nutrients, root phytoalexins, and microbial communities in rhizosphere and root-affected soil were analyzed. Catch crop amendments but not microbial inoculations, significantly altered soil nutrients. Root length increased significantly under CCM, and in tendency in *Tagetes* and SynC. Phytoalexin contents were lowest in *Tagetes* and highest in CCM, both differing from the control in specific compounds. Microbiome analysis revealed that catch crops strongly modulated fungal communities in rhizosphere and root-affected soil, favoring potentially beneficial *Linnemannia* and *Mortierella*, while microbial inoculations predominantly modulated bacterial/archaeal rhizosphere communities. Our results suggest that catch crops and microbial inoculants induced distinct shifts in soil–plant–microbe interactions under replanting conditions.

## Teaser

Catch crop amendments and microbial inoculants induce distinct alterations in the rhizosphere and soil microbiome of apple grown under replanting conditions, favoring potential plant beneficials, and alter biochemical plant responses.

## Introduction

The soil microbiome is a key component of healthy soils and increasingly considered important in agricultural cultivation systems and practices (Fierer 2017). Soil microbes drive essential processes and ecosystem functions, including soil formation, nutrient cycling, and plant health (Chenu and Cosentino 2011, Banerjee and van der Heijden 2022). Plants recruit soil microorganisms to form their rhizosphere microbiome, which can be seen as a primary defense line against soilborne pathogens (Philippot et al. 2013, Nwokolo et al. 2021). For agricultural soils under intensive crop cultivation, maintaining soil health and productivity depends on the effective management of the bulk soil and rhizosphere microbiome (van der Heijden and Wagg 2013). Monocropping alters the rhizosphere microbiome, often reducing plant performance due to negative plant–soil feedback (Berihu et al. 2023, Kaloterakis et al. 2025). This phenomenon arises from plant metabolites that accumulate in the soil or pathogenic microbes that proliferate under monocropping conditions (van der Putten et al. 2013, Wang et al. 2024a).

An example of negative plant–soil feedback is apple replant disease (ARD), which affects apple orchards and tree nurseries worldwide, reducing productivity and hindering replanting efforts (Winkelmann et al. 2019, Somera and Mazzola 2022). ARD symptoms include vestigial root development and reduced plant growth (Henfrey et al. 2015, Mahnkopp et al. 2018). ARD is induced at the root–soil interface and linked to changes in the rhizosphere and bulk soil microbiome, including the accumulation of plant pathogenic microorganisms (Tewoldemedhin et al. 2011, Popp et al. 2020, Balbín-Suárez et al. 2021, Hauschild et al. 2024). Additionally, affected plants exhibit biochemical responses, such as elevated phytoalexin levels and changes in root exudates, indicating compromised plant resilience (Wittenmayer and Szabó 2000, Weiß et al. 2017, Busnena et al. 2025). ARD mitigation traditionally relied on chemical soil fumigation, which not only disrupts the soil microbiome (Somera and Mazzola 2022) but also has further adverse environmental side effects, accounting for an urgent need for alternatives (Winkelmann et al. 2019, Sharma et al. 2020a). Various approaches have been explored, but no universal solution seems to exist due to the disease complexity (Somera and Mazzola 2022). Most studies investigating ARD countermeasures assessed plant growth response (Utkhede et al. 2001, Braun et al. 2010, Duan et al. 2022), and several also examined microbiome alterations (Mazzola et al. 2015, Yim et al. 2017, Li et al. 2018). Understanding microbiome shifts in conjunction with plant biochemical responses is crucial for developing effective management strategies for negative plant–soil feedback. So far, the universal success of most proposed management options has failed because their underlying mechanisms, including plant–microbe interactions are poorly understood. Two promising approaches to restore the diversity and functionality of the disturbed soil microbiome involve the amendment of catch crops or the application of microbial inoculants.

Catch crops, typically grown between successive main crop plantings, can enhance perennial plant health by improving nutrient availability and modifying soil microbial communities to suppress pathogens (De Corato 2020, Seitz et al. 2024). Catch crops are traditionally grown as monocultures that offer limited functional diversity (Gentsch et al. 2020). However, incorporating diverse plant species may enhance microbial interactions and soil health benefits (Freund et al. 2021). Yim et al. (2016, 2017) showed that incorporating *Tagetes patula* as a catch crop improved apple plant growth in ARD-affected soil. More recent studies suggest that multispecies catch crop mixtures (CCMs) provide broader ecological benefits by increasing plant biodiversity, offering diverse nutrients and ecological niches, and increasing the abundance and activity of beneficial microbes (Gentsch et al. 2020, Freund et al. 2021).

Another promising approach is the inoculation of soil or plants with plant-beneficial microbes, which can enhance plant growth by improving nutrient uptake, producing plant hormones, or inducing systemic resistance against pathogens (Finkel et al. 2017, Berg et al. 2021, Compant et al. 2025). In the rhizosphere, microbial inoculants can also reshape the native microbiome (Mawarda et al. 2020, Behr et al. 2023). While single-species inoculants have been widely used, synthetic microbial communities, i.e. combinations of multiple beneficial strains, are gaining attention due to their potential synergistic effects. These consortia can enhance nutrient cycling, suppress pathogens, and support plant health more effectively than single-strain inoculants (Finkel et al. 2017, Bradáčová et al. 2019, De Souza et al. 2020, Gonҫalves et al. 2023).

**Figure 1.**
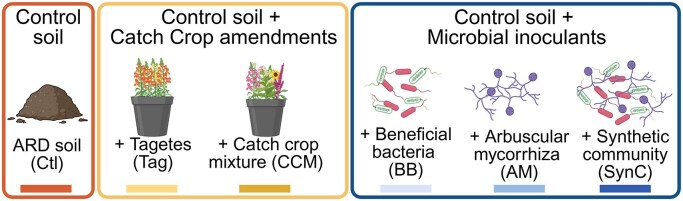
Overview of the different treatments included in the present study. Untreated ARD-affected soil served as control (Ctl). Two treatments testing the amendment of catch crops were *T. patula* (Tag) as a single catch crop and a catch crop mixture (CCM). Three treatments testing microbial inoculants included inoculation of plant-beneficial bacteria *Pseudomonas* sp. RU47 and *B. atrophaeus* ABi05 (BB), arbuscular mycorrhiza *R. irregulare* and *F. mosseae* (AM), and a synthetic community (SynC) comprising BB and AM. Figure created with biorender.com

In this study, we examined how soil microbiomes associated with young apple plants grown in ARD-affected soil respond to the incorporation of catch crop amendments or inoculation of microbial inoculants in a greenhouse experiment (Fig. 1). Five treatments were compared to plants grown in untreated ARD-affected soil (Ctl). We tested two catch crop amendments: incorporating either a single plant species, *T. patula* (Tag), or a diverse crop mixture containing 12 plant species (CCM). Additionally, we tested three microbial inoculants, (i) beneficial bacteria (BB; *Pseudomonas* sp. RU47 and *Bacillus atrophaeus* ABi05), (ii) arbuscular mycorrhiza fungi (AM; *Rhizoglomus irregulare* and *Funneliformis mosseae*), and (iii) a synthetic microbial community (SynC) comprising BB and AM. The microbial inoculants were selected because of their complementary plant growth-promoting traits. RU47 possesses several plant-beneficial traits, i.e. solubilization of phosphate, production of indole-3-acetic acid, siderophores, HCN and activity of hydrolytic enzymes, and growth promotion of different crops was reported (Adesina et al. 2009, Kuzmanović et al. 2018, Schreiter et al. 2018, Eltlbany et al. 2019). For ABi05 or closely related *B. velezensis* strains, the phytostimulatory effect, the potential to suppress several bacterial and fungal plant pathogens, and the ability to produce siderophores and hydrolytic enzymes (cellulase, chitinase, β-1,3-glucanase, and protease) were shown *in vitro* (Borriss et al. 2016, Sella et al. 2015, Behr et al. 2023). The symbiosis between plants and AM can improve plant growth and enhance the tolerance toward abiotic and biotic stresses (Alagna et al. 2020). In exchange for plant photosynthetic carbon, AM provide plants with nutrients, particularly phosphate and nitrogen, and water and other elements by extending the root zone through hyphae formation (Smith and Smith 2011).

We hypothesized that catch crop amendments and microbial inoculants improve apple plant performance in ARD-affected soil and modulate the soil microbiome. We tested our hypothesis by analyzing soil nutrient contents, measuring root phytoalexins as indicators of biochemical plant responses, and examining microbiome composition in rhizosphere and root-affected soil. Additionally, spatial differences were accounted for by quantifying bacterial inoculants and phytoalexins across different root parts.

## Materials and methods

### Soils and catch crop amendments

Topsoil (0–20 cm) from the experimental reference site in Heidgraben, Germany (53° 41′ 57.5″ N, 9° 40′ 59.6″ E) was collected in June 2021 and has been characterized as Entic Podzol by the Status of the World’s Soil Resources (FAO and ITPS 2015). ARD soil was collected from four plots on which ARD had been induced by repeated planting of rootstock “Bittenfelder Sämling” since 2009, described in detail by Mahnkopp et al. (2018). The soil was homogenized by sieving through a 2 mm mesh and stored at 4°C until the experiment was set up.

Soils for *Tagetes* (Tag) or CCM amendment were prepared as follows: *T. patula* 'Nemamix' (Deutsche Saatveredelung AG, Steinhorst, Germany) was sown into ARD soil with 1.00 g m^−2^. CCM was sown into ARD soil with 2.14 g m^−2^. The CCM comprised 12 plant species at different proportions: *Vicia villosa* (20%), *Sorghum sudanese* (15%), *Avena strigosa* (10%), *Brassica juncea* 'Terrafit' (10%), *T. patula* 'Nemamix' (10%), *Brassica carinata* (5%), *Camelina sativa* (5%), *Melilotus albus* (5%), *Raphanus sativus* 'Black Jack' (5%), *Raphanus sativus* 'Deeptill' (5%), *Sinapis alba* (5%), and *Trifolium incarnatum* 'Linkarus' (5%) (kindly provided by Deutsche Saatveredelung AG, Steinhorst, Germany). Tag and CCM were grown in a greenhouse for three months at 19.9 ± 2.0/18.5 ± 0.8°C (day/night) and a relative humidity of 72.2 ± 10.9%. After the plants reached the flowering stage, they were removed from the pots, and their roots and shoots were cut into 3–4 cm pieces. We incorporated the chopped plant debris thoroughly into the soil and stored it for seven weeks in the greenhouse under the abovementioned conditions to initiate organic matter turnover until the experiment was set up.

### Microbial inoculants

Microbial inoculants were used in three of the five treatments: BB, AM, and a synthetic community (SynC) comprising BB and AM. Treatment BB included strain *B. atrophaeus* ABi05, provided as a ready-to-use spore suspension [4.5 × 10^10^ colony forming units (CFU) ml^−1^] by ABiTEP GmbH (Berlin, Germany, DSM29418) and cells of the strain *Pseudomonas* sp. RU47 (DSMZ, Braunschweig, Germany, DSM117411), prepared as described by Hauschild et al. (2024). Briefly, overnight cultures of RU47 were grown in Luria–Bertani (LB) broth (Carl Roth GmbH, Karlsruhe, Germany) supplemented with ampicillin (100 µg ml^−1^, SERVA Electrophoresis GmbH, Heidelberg, Germany), chloramphenicol (30 µg ml^−1^, Sigma-Aldrich GmbH, Taufkirchen, Germany), and rifampicin (50 µg ml^−1^, SERVA). Overnight cultures were centrifuged and washed in a sterile 0.85% NaCl solution. Finally, the inoculation suspension was prepared by resuspending the pellet and adjusting the optical density corresponding to 1 × 10^7^ CFU ml^−1^ with sterile water. Bacterial inoculants, ABi05 and RU47, were rifampicin-resistant mutants, which allowed their detection by CFU counting using selective media. For treatment AM, spores of *R. irregulare* and *F. mosseae* were provided as a ready-to-use powder by INOQ GmbH (“INOQ Advantage”; Schnega, Germany).

### Plant material

The apple rootstock M.26 was used as a model plant in the greenhouse due to its ARD susceptibility. M.26 was propagated *in vitro*, as described in detail by Rohr et al. (2020). Briefly, Murashige and Skoog (MS) medium (Murashige and Skoog 1962) containing 3% (w/v) sucrose, 4.4 µM 6-benzylaminopurine, and 0.5 µM indole-3-butyric acid was used to multiply shoot cultures. To induce rooting, single shoots were transferred to 1/2 MS with 2% (w/v) sucrose and 4.92 µM indole-3-butyric acid. To acclimatize the rooted plants to greenhouse conditions, they were transferred to a commercial peat substrate (Steckmedium, Klasmann-Deilmann GmbH, Geeste, Germany) one month before starting the experiment.

### Experimental set-up

At the experimental set-up, the peat substrate was carefully removed from the acclimatized M.26 plants. For each of the six treatments, six biological replicates of 700 ml soil per pot at a density of 1.1 g cm^−3^ soil and a final volumetric water content of 20% were set up, each with one plant, resulting in 36 units in total. All soils were fertilized with 2 g l^−1^ Osmocote exact 3–4 M (16% N+ 9% P_2_O_5_+ 12% K_2_O+ 2% MgO; ICL Deutschland, Nordhorn, Germany). Plants were potted in ARD-affected soil either untreated (Ctl) or treated with the following amendments: for treatments Tag and CCM, the prepared soils with the decomposed catch crop plant material were filled in pots and planted with M.26. For treatment BB, spores of ABi05 were prediluted in 50 ml sterile water before incorporation into Ctl soil at a density of 1 × 10^7^ ABi05 spores cm^−3^ soil using a sterile shovel. Pots were filled with the ABi05-inoculated soil. The inoculation of plants with RU47 was performed by root-dipping for 30 min in sterile aluminum boxes filled with 300 ml inoculation suspension. After dipping, the RU47 inoculated plants were planted into the soil with ABi05 spores, resulting in treatment BB. Accordingly, plants for Ctl, Tag, CCM, and AM were dipped for 30 min in sterile water before planting into uninoculated soil. For treatment AM, the provided spore powder was premixed in 30 ml sterile sand and incorporated into Ctl soil at a density of 0.1 mg AM spores cm^−3^ soil using a sterile shovel, filled into pots and planted with M.26. For the treatment SynC, BB and AM were combined: 1 × 10^7^ ABi05 spores cm^−3^ soil and 0.1 mg AM spores cm^−3^ soil were incorporated into the soil and RU47 was applied by 30 min root-dipping in 1 × 10^7^ CFU ml^−1^ inoculation suspension before planting in the ABi05 and AM inoculated soil.

Pots of the six different treatments were placed in trays with fiber mats to facilitate a steady water supply, and plants were watered evenly by wetting the fiber mats every other day. Plants were cultivated for seven weeks, from 19 October to 30 November 2021, in a greenhouse chamber with an average humidity of 54.8 ± 9.2% at a mean temperature of 20.9 ± 1.4/ 17.9 ± 0.8°C (day/night) and a 16 h photoperiod. If the photosynthetic active radiation was below 182 µmol m^−2^ s^−1^, additional light was provided by high-pressure sodium lamps (MASTER SON-T PIA Plus, Philips Lighting, Eindhoven, The Netherlands). To measure soil dehydrogenase activity, CO_2_ respiration, and C/N ratios, ~400 ml remaining soil of each treatment was filled in unsealed plastic bags, stored overnight at 4°C, and processed as described in File S1.

### Sampling and sample processing

After seven weeks of growth in the greenhouse, we harvested all plants in one final sampling (Fig. S1). The plants were carefully removed from the pots. Shoots and roots were separated using sterile scissors. For each shoot, length and fresh mass were measured. The shoot length increase for each plant was calculated as the difference in shoot length at sampling and experimental set-up. The soil remaining in the pots, termed root-affected soil, was mixed to get a homogenous sample. A subsample was transferred to 2 ml reaction tubes; the remaining soil was transferred into plastic bags and stored at −20°C for subsequent total microbial DNA extraction and soil nutrient analysis. From each plant, loosely adhering soil was removed from the roots by gentle shaking, leaving the root system with the attached rhizosphere soil. The root system was split into halves and processed as follows: one half was carefully brushed to harvest the rhizosphere. Rhizosphere samples were stored at −20°C until microbial DNA extraction. The brushed roots were weighed and carefully wrapped in moist paper towels until root morphology measurements were taken. The other half of the root was separated into three parts: the upper basal roots close to the stem, the lower basal roots as the middle part of the root system, and the root tips, i.e. the last 2–3 cm of the roots (Fig. S1). The rhizosphere was harvested from each part by carefully brushing the roots and processed immediately to quantify the bacterial inoculants by selective plating. The brushed root parts of each plant were weighed and immediately frozen in liquid nitrogen and stored at −80°C until extraction of phytoalexins.

### Root morphological analysis

To analyze root morphology, one half of each root system (Fig. S1) sampled as described above was scanned at 720 dots per inch with 35 μm resolution using a flatbed scanner (EPSON perfection V700, Seiko Epson Corporation, Nagano, Japan). WinRhizo 2019 software (Regent Instruments, Québec City, Canada) was used to analyze the root traits. Root length was measured in four diameter classes divided into 500 µm intervals ranging from <500 µm to ≥1.5 mm. The final total root length was estimated based on the total root fresh mass of both root halves. The procedure of estimating total root length from subsamples has been validated previously (Hauschild et al. 2024).

### Soil nutrient analysis

The content of soil macro- and micronutrients, including Fe, K, Mg, P, NO_3_-N, NH_4_-N, B, Cu, Mn, Na, and Zn, as well as soil pH, dry matter content (DM), and mineral N (N_min_; NO_3_/NH_4_ ratio) were analyzed according to certified VDLUFA (Association of German Agricultural Analytic and Research Institutes, Speyer, Germany) procedures at AGROLAB GmbH (Leinefelde-Worbis, Germany).

### Detection and quantification of phytoalexins in roots via GC–MS

Root phytoalexins were individually analyzed for the three root parts (upper and lower basal roots and root tips) (Fig. S1). The sampled root parts were lyophilized and homogenized to a fine powder for 1 min in a mixer mill (29 Hz, MM400, Retsch, Haan, Germany). Detailed protocols used for the extraction and quantification of phytoalexins using GC–MS were published by Busnena et al. (2021). Briefly, the root powder was extracted with 1 ml methanol supplemented with 25 µg 4-hydroxybiphenyl, serving as internal standard, by vigorous vortexing (2,700 rpm, 20 min, Vortex Genie2, Scientific Industries, Bohemia, NY, USA). The extracts were centrifuged (13,400 × *g*, 10 min) and the supernatants were airstream-dried before the residues were redissolved in 1 ml dichloromethane: chloroform (1:1, v/v). Centrifugation (13,400 × *g*, 10 min) and airstream-drying of the supernatants were repeated and the residues were redissolved in 200 µl ethyl acetate. After another centrifugation step (13,400 × *g*, 10 min), the supernatants were transferred to GC–MS vials with a glass inlet. After removal of ethyl acetate by airstream-drying, the residues were redissolved in 50 µl *N*-methyl-*N*-(trimethylsilyl)-trifluoroacetamide (MSTFA; ABCR, Karlsruhe, Germany) and silylated for 30 min at 60°C. GC–MS analysis of the silylated samples was done at 70°C for 3 min, 70–310°C for 24 min (10°C ­min^−1^), 310°C for 5 min, with a helium flow of 1 ml min^−1^, an injection volume of 1 µl and a split ratio of 1:10. For relative quantification of the individual compounds, the added internal standard 4-hydroxybiphenyl was employed, enabling a relative quantitative comparison of the levels of phytoalexin contents in all samples. A set of even-numbered hydrocarbons (C_14_–C_32_) was coinjected to calculate the retention indices using linear extrapolation as previously described (Busnena et al. 2021).

### Detection of bacterial inoculants

For treatments Ctl, BB, and SynC, CFU counts of RU47 and ABi05 were determined for the rhizosphere from each root part and root-affected soil, with Ctl as a non-inoculated negative control (Fig. S1). For this, 0.1 g of rhizosphere from each of the three root parts was diluted in 900 µl with sterile 0.85% NaCl and vigorously mixed by vortexing for 1 min. Serial dilutions were plated in duplicate onto Reasoner’s 2 agar (R2A, Merck KGaA, Darmstadt, Germany) supplemented with cycloheximide (100 µg ml^−1^, SERVA) and rifampicin (50 µg ml^−1^) to cultivate ABi05 and on King’s B agar (Carl Roth GmbH) supplemented with cycloheximide (100 µg ml^−1^), ampicillin (100 µg ml^−1^), chloramphenicol (30 µg ml^−1^), and rifampicin (50 µg ml^−1^) to cultivate RU47. To calculate the ratio of ABi05 spores and vegetative cells, suitable dilutions were heat shock-treated at 90°C for 10 min to inactivate all vegetative cells, leaving only viable spores. The samples were cooled on ice for 10 min and plated on R2A media as described above. CFUs were counted after incubation at 28°C for 48 h.

### Microbial community DNA extraction and amplicon sequencing

Microbial community DNA was extracted from 0.5 g of the root-affected soil and the rhizosphere (Fig. S1) using the Fast-Prep-24-bead-beating system and the FastDNA Spin Kit for Soil (MP Biomedicals, Eschwege, Germany). Afterwards, DNA extracts were purified with the Geneclean Spin Kit (MP Biomedicals) according to the companies’ protocols. The quality of the cleaned DNA extracts was checked on a 0.8% agarose gel and quantified spectrophotometrically (Nanodrop, Thermo Fisher, Waltham, USA). Purified DNA extracts served as a template to amplify the bacterial/archaeal *16S rRNA* gene (V3–V4 region) and the fungal ITS2 region, using primer pairs 341F/806R (Sundberg et al. 2013) and gITS7/ITS4 (White et al. 1990, Ihrmark et al. 2012), respectively. Amplicon sequencing, including polymerase chain reaction (PCR) amplification, quality control, library preparation, and sequencing on an Illumina MiSeq v2 PE250 (Illumina Inc., San Diego, USA) was performed at the sequencing service provider Novogene Co. (Cambridge, UK) according to the companies’ standard procedures. For some treatments, only five replicates were sequenced since for some samples (root-affected soil: bacterial/archaeal SynC-2; fungal Ctl-2, Tag-5, CCM-2, BB-5, AM-2, and SynC-5; rhizosphere: fungal Ctl-2, Tag-5, CCM-5, BB-2, AM-5, and SynC-2) the PCR amplification step failed.

### Bioinformatic and statistical analyses

Bioinformatic analysis was performed in R version 4.3.1 (R Core Team 2023) and R studio version 4.3.2 (R Studio Team 2023). Statistical evaluation of the treatment effect on soil microbial activity, plant growth, and soil nutrient data was done by one-way analysis of variance (ANOVA) or two-way ANOVA for the effect of treatment and root part on CFU counts and root phytoalexin content. *Post hoc* Tukey’s HSD test using the “agricolae” package (De Mendiburu and Yaseen 2020) was carried out after the null hypothesis for normality was accepted (*p* > 0.05) for each dataset using the Shapiro–Wilks test. Raw amplicon sequences obtained from *16S rRNA* gene and ITS fragment sequencing were processed and classified using DADA2 version 1.12.1 (Callahan et al. 2016). The “filterAndTrim” function was used for quality trimming and filtering, and two expected errors per read were allowed. Trimmed sequences were error-corrected, denoised, and chimeras were removed. Using the cleaned reads, amplicon sequence variants (ASVs) were classified using the SILVA database version 138 (Quast et al. 2013) for ASVs from the *16S rRNA* gene dataset and the UNITE general FASTA release for Fungi 2 version 10.0 (Abarenkov et al. 2024) for ASVs from the ITS fragment dataset. It resulted in 82,839 unique ASVs for the *16S rRNA* gene and 12,763 unique ASVs for the ITS fragment. ASVs not assigned at the phylum level or assigned as chloroplast or mitochondria were removed from the datasets. ASVs were named according to the closest taxonomic rank curated in the database. Bacterial ASVs annotated as *Allorhizobium–Neorhizobium–Pararhizobium–Rhizobium* were renamed to *Allo_Neo_Para_Rhizobium* and *Burkholderia–Caballeronia–Paraburkholderia* were renamed to *Para_Burkholderia–Caballeronia*. Cleaned ASV, taxonomy, and metadata tables of datasets were imported to the “phyloseq” package (McMurdie and Holmes 2013). All subsequent analyses, except differential abundance testing, were performed on the rarefied datasets following recommendations by Schloss (2024). Samples were rarefied at the lowest number of sequences across all samples (*16S rRNA* gene: 36,692 sequence reads; ITS fragment: 49,725 sequence reads). Rarefaction curves reached a saturation plateau for all samples, indicating that sufficient sequences were obtained to cover most of the samples’ diversity (Fig. S2). Species richness (Chao1), evenness (Pielou), and diversity (Shannon) indices were calculated using the “vegan” package (Oksanen et al. 2020) and displayed in violin charts using “ggplot2” (Wickham 2016). To determine significant differences, Kruskal–Wallis and pairwise *post hoc* Wilcoxon tests with Benjamini–Hochberg correction were employed using the “agricolae” package (De Mendiburu and Yaseen 2020). To analyze beta-diversity, Bray–Curtis dissimilarity indices were calculated to create a distance matrix based on square root transformed count data. We used Principal Coordinate Analysis (PCoA) to visualize dissimilarities between the microbial communities. To test for statistical differences between microhabitats and treatments, permutational multivariate ANOVA (PERMANOVA) with 10,000 permutations and pairwise Adonis was performed using “vegan” (Oksanen et al. 2020) and “pairwise.adonis” (Martinez Arbizu 2020) packages. Taxa with differential abundance between the Ctl and each treatment were identified based on the negative binomial distribution implemented in DeSeq2 using raw count data (Love et al. 2014). Taxa were considered differentially abundant if adjusted *p*-values (*p-adj*) were <0.05 after Benjamini–Hochberg correction. To display the microbial community composition, data were transformed to relative abundance and visualized using the packages “ggplot2” (Wickham 2016) and “pheatmap” (Kolde 2019). To assess the presence of *Pseudomonas* sp. RU47 and *Bacillus athropaeus* ABi05 inoculants among the retrieved ASVs, the ASV sequences identified as *Pseudomonas* or *Bacillus* at the genus level were compared with the RU47 genome sequence (accession number CP022411, GenBank) or partial *16S rRNA* gene sequences of *B. atrophaeus* type strain sequences available in the NCBI database, respectively.

## Results

### Root growth and soil nutrients were modified by catch crop amendments but not microbial inoculants

After growth in the differently treated soils for seven weeks, root length was significantly (*p-adj* = 0.026) higher for plants grown in CCM (2,079 ± 678 cm), compared to the Ctl (1,325 ± 255 cm) (Fig. S3A). The large variation in plant growth within treatments led to a high standard deviation, making it difficult to detect significant changes in root and shoot growth. Although root systems of plants grown in Tag (1,720 ± 388 cm) and SynC (1,472 ± 318 cm) were longer compared to the Ctl, these differences were not significant (Tag: *p-adj* = 0.518; SynC: *p-adj* = 0.986). Shoot length increase showed no significant differences among the treatments (*p* = 0.450). Still, plants in Ctl exhibited the lowest shoot length increase (2.15 ± 0.48 cm), while shoots of plants grown in treated soils performed slightly better (Tag: 2.93 ± 0.49 cm; CCM: 2.72 ± 0.36 cm; BB: 2.88 ± 0.87 cm; AM: 2.57 ± 0.19 cm; SynC: 2.55 ± 0.20 cm) (Fig. S3B).

Measurements of soil nutrient contents after 42 days revealed that changes in soil physicochemical parameters were mainly induced by catch crop amendments (Tag, CCM), while no changes were observed in the inoculated soils (except for Fe). In detail, Na contents in soils amended with Tag and CCM were significantly higher compared to the Ctl (Table 1). Also, N_min_ and Zn were higher while Fe was lower in Tag and CCM than in the Ctl. Additionally, the Tag amended soil had higher K, Mg, NH_4_-N contents, and the pH value was increased compared to the Ctl. In soil amended with CCM, the Mn content was almost twice as high as in the Ctl. Contrarily, the inoculation of BB, AM, or SynC did not induce significant changes compared to the Ctl, except for a significantly lower Fe content in treatment BB.

**Table 1.** Characteristics of root-affected soil of apple rootstock M.26 grown for 42 days in ARD soil (Ctl) that was treated with *T. patula* (Tag), a catch crop mixture (CCM), beneficial bacteria (BB), arbuscular mycorrhiza (AM), or a synthetic community comprising BB and AM (SynC). Nutrient analysis was done using certified procedures according to VDLUFA at AGROLAB GmbH (Leinefelde-Worbis, Germany). Average values and standard deviation of six replicates are depicted. Different letters indicate significant differences (*p* < 0.05) among treatments according to ANOVA and Tukey’s HSD test. Values in bold highlight significant differences between the respective treatments and Ctl. DM: dry matter

	Ctl	Tag	CCM	BB	AM	SynC
**pH**	4.93 ± 0.19 bc	**5.22 ± 0.12 a**	5.17 ± 0.12 ab	4.98 ± 0.13 abc	4.85 ± 0.10 c	5.02 ± 0.18 abc
**DM** (%)	93.06 ± 1.14 ab	95.02 ± 0.90 a	94.77 ± 1.31 a	91.85 ± 2.63 ab	91.21 ± 1.54 b	93.21 ± 2.56 ab
**N_min_** (kg ha^−1^)	1031 ± 159 b	**1405 ± 338 a**	**1497 ± 318 a**	1170 ± 248 ab	1048 ± 349 ab	1147 ± 276 ab
Macronutrients (mg 100 g^−1^ soil)						
**Fe**	24.40 ± 1.63 a	**21.38 ± 1.84 b**	**21.27 ± 0.97 b**	**21.58 ± 1.25 b**	23.23 ± 1.60 ab	22.07 ± 1.23 ab
**K**	20.68 ± 4.40 b	**33.47 ± 7.01 a**	24.05 ± 6.88 b	18.20 ± 2.42 b	17.57 ± 4.23 b	20.75 ± 5.68 b
**Mg**	7.62 ± 0.93 bc	**10.35 ± 1.06 a**	8.93 ± 1.28 ab	7.13 ± 0.55 c	6.77 ± 0.69 c	7.50 ± 0.74 bc
**P**	18.48 ± 1.46 ab	21.58 ± 2.97 a	18.07 ± 2.24 b	17.63 ± 0.86 b	17.28 ± 1.07 b	18.77 ± 2.18 ab
**NO_3_-N**	17.77 ± 3.51 a	21.83 ± 5.42 a	24.68 ± 5.42 a	18.52 ± 4.29 a	17.03 ± 4.52 a	18.56 ± 4.00 a
**NH_4_-N**	5.09 ± 1.09 b	**9.92 ± 2.30 a**	9.09 ± 2.29 ab	7.09 ± 2.19 ab	5.74 ± 3.68 ab	7.38 ± 2.32 ab
Micronutrients (mg kg^−1^ soil)						
**B**	0.37 ± 0.07 ab	0.49 ± 0.05 a	0.39 ± 0.10 ab	0.34 ± 0.06 b	0.33 ± 0.11 b	0.41 ± 0.10 ab
**Cu**	3.90 ± 0.24 a	3.77 ± 0.59 a	4.00 ± 0.47 a	3.43 ± 0.71 a	4.13 ± 0.46 a	3.78 ± 0.40 a
**Mn**	13.17 ± 0.75 b	13.67 ± 1.03 b	**23.00 ± 1.10 a**	12.67 ± 1.37 b	13.33 ± 1.37 b	13.67 ± 1.21 b
**Na**	23.33 ± 4.55 b	**65.33 ± 11.74 a**	**72.50 ± 16.40 a**	24.17 ± 3.19 b	24.67 ± 2.58 b	25.50 ± 4.09 b
**Zn**	11.80 ± 0.51 bc	**13.25 ± 0.41 a**	**12.90 ± 0.30 a**	11.77 ± 0.50 bc	11.37 ± 0.45 c	12.13 ± 0.39 b

### The composition and concentration of root phytoalexins differed among treatments

Two-way ANOVA revealed that total phytoalexin concentrations were highly different between the root parts (upper and lower basal roots and root tips) (*p* = 2.78E-15) but not between treatments (*p* = 0.083). Therefore, the concentrations of individual phytoalexin compounds were analyzed separately for each root part. In total, 19 different phytoalexin compounds were identified (Table 2). In all root parts, 2-hydroxy-4-methoxydibenzofuran (RI_2131), noraucuparin (RI_2121), 3-hydroxy-5-methoxybiphenyl (RI_1956), hydroxyeriobofuran isomer 2 (RI_2331), and noreriobofuran (RI_2259) were the compounds with highest concentrations (>100 µg g^−1^ DM). CCM roots had the highest phytoalexin concentrations, and significant differences for certain compounds were mainly observed between CCM and Tag as follows. In the lower basal roots, the concentrations of noraucuparin (RI_2121), methoxyeriobofuran isomer 1 (RI_2245) and hydroxyeriobofuran isomer 1 (RI_2280) were significantly higher in roots of CCM than in Tag (Table 2). Interestingly, noreriobofuran (RI_2259) was absent in the lower basal roots of Tag and significantly higher in Ctl and all inoculation treatments, while its concentration was moderate in CCM. The same tendency was observed in the root tips, although not significant. In the lower and upper basal roots, 3-hydroxy-4,5-dimethoxybiphenyl (RI_2037) was significantly lower in Ctl roots than in CCM. In the upper basal roots, the eriobofuran (RI_2228) concentration was significantly higher in Ctl, CCM, and AM than in Tag and SynC. The methoxyeriobofuran isomer 1 (RI_2245) concentration was significantly higher in the upper basal roots of CCM than of Tag, where it was absent, and SynC, where its concentration was very low. We did not identify significant differences between the Ctl and the inoculation treatments (BB, AM, and SynC) for any of the 19 compounds, except for a significantly lower eriobofuran (RI_2228) concentration in SynC compared to Ctl.

**Table 2.** Concentration of phytoalexins in three parts of the root system of apple rootstock M.26 grown for 42 days in ARD soil (Ctl) that was treated with *T. patula* (Tag), a catch crop mixture (CCM), beneficial bacteria (BB), arbuscular mycorrhiza (AM), or a synthetic community comprising BB and AM (SynC). Average values and standard deviation of six replicates are depicted. Different letters indicate significant differences (*p* < 0.05) among treatments according to ANOVA and Tukey’s HSD test and missing letters indicate no significant differences. Significant differences compared to Ctl are highlighted in bold. RI: retention index of respective phytoalexin. RI_1956: 3-hydroxy-5-methoxybiphenyl; RI_2037: 3-hydroxy-4,5-dimethoxybiphenyl; RI_2090: aucuparin, RI_2121: noraucuparin; RI_2131: 2-hydroxy-4-methoxydibenzofuran; RI_2173: eriobofuran_isomer1; RI_2228: eriobofuran; RI:2245: methoxyeriobofuran isomer 1; RI_2259: noreriobofuran; RI_2280: hydroxyeriobofuran isomer 1; RI_2331: hydroxyeriobofuran isomer 2; RI_2346: eriobofuran isomer 2; Low: 3,5-dihydroxybiphenyl, noreriobofuran isomer 1, hydroxynoreriobofuran isomer 1, hydroxynoreriobofuran isomer 2, hydroxynoreriobofuran isomer 3, methoxyeriobofuran isomer 4, hydroxyeriobofuran isomer 3; and PA_total: total phytoalexin concentration.

		Phytoalexin concentration (µg g^−1^ root DM)													
	Treat	RI_1956	RI_2037	RI_2090	RI_2121	RI_2131	RI_2173	RI_2228	RI_2245	RI_2259	RI_2280	RI_2331	RI_2346	Low (<10)	PA_total
**Root tips**	**Ctl**	197 ± 147	2.77 ± 6.78	14.55 ± 11.2	172 ± 152	582 ± 137	22.6 ± 19.8	20.6 ± 12.9	0.00	61.0 ± 40.3	9.66 ± 7.33	141 ± 54.5	47.72 ± 15.5	9.10	1280 ± 509
	**Tag**	97.2 ± 37.7	10.3 ± 8.90	15.97 ± 6.0	104 ± 32.3	394 ± 64.5	27.3 ± 15.2	11.8 ± 9.65	0.00	0.00	9.54 ± 10.1	96.9 ± 35.8	62.81 ± 20.7	21.76	852 ± 169
	**CCM**	197 ± 87.1	22.2 ± 20.8	46.34 ± 21.2	444 ± 93.7	507 ± 213	56.5 ± 17.2	34.3 ± 16.9	1.04 ± 2.55	68.0 ± 86.6	31.8 ± 35.1	97.0 ± 59.6	61.94 ± 28.7	28.97	1596 ± 419
	**BB**	147 ± 123	6.54 ± 13.2	29.76 ± 28.3	210 ± 165	527 ± 180	35.3 ± 23.5	29.8 ± 25.6	0.00	67.8 ± 95.1	8.98 ± 9.15	154 ± 89.4	63.97 ± 62.5	32.53	1312 ± 608
	**AM**	145 ± 136	5.66 ± 13.9	24.24 ± 32.3	282 ± 502	569 ± 190	29.9 ± 28.4	29.8 ± 20.3	0.51 ± 1.25	113 ± 167	7.61 ± 15.5	114 ± 70.4	46.43 ± 17.8	25.10	1392 ± 1 198
	**SynC**	84.2 ± 26.9	7.90 ± 19.4	15.81 ± 18.7	185 ± 161	455 ± 91.0	25.1 ± 18.4	17.7 ± 23.6	0.50 ± 1.22	85.6 ± 104	14.3 ± 24.2	123 ± 78.0	60.53 ± 26.4	33.07	1108 ± 522
**Lower basal roots**	**Ctl**	103 ± 45.6	**9.29 ± 8.61 b**	31.88 ± 29.7	216 ± 79.1 ab	261 ± 65.7	28.2 ± 14.1 ab	29.5 ± 20.1	1.22 ± 1.40 ab	**139 ± 82.4 a**	6.65 ± 4.86 ab	172 ± 52.2	36.67 ± 11.0	49.91	1084 ± 231
	**Tag**	102 ± 55.2	22.9 ± 9.21 ab	12.26 ± 4.2	124 ± 50.1 b	205 ± 61.2	30.3 ± 10.8 ab	10.2 ± 3.16	0.62 ± 0.99 b	**0.00 b**	1.09 ± 1.70 b	97.8 ± 49.9	47.09 ± 36.0	26.01	679 ± 196
	**CCM**	167 ± 107	**34.1 ± 23.9 a**	39.00 ± 13.4	369 ± 233 a	223 ± 118	61.1 ± 38.3 a	29.0 ± 10.7	3.93 ± 1.83 a	57.9 ± 142 ab	13.0 ± 12.1 a	111 ± 82.5	29.14 ± 13.8	50.34	1187 ± 723
	**BB**	74.8 ± 38.2	7.47 ± 4.25 b	37.13 ± 36.5	178 ± 65.6 ab	201 ± 96.7	22.3 ± 12.9 b	25.6 ± 15.2	1.32 ± 2.17 ab	105 ± 92.3 a	5.51 ± 3.60 ab	126 ± 47.5	43.52 ± 42.5	51.85	880 ± 304
	**AM**	75.1 ± 50.3	10.4 ± 14.2 b	27.97 ± 20.6	235 ± 183 ab	190 ± 80.9	28.1 ± 19.3 ab	26.3 ± 15.0	2.20 ± 2.20 ab	182 ± 92.9 a	4.96 ± 3.45 ab	98.8 ± 45.9	27.68 ± 13.2	44.28	954 ± 469
	**SynC**	76.3 ± 30.2	9.37 ± 9.19 b	23.37 ± 5.8	197 ± 43.2 ab	219 ± 67.4	26.0 ± 10.2 ab	23.7 ± 6.97	2.16 ± 1.83 ab	104 ± 91.2 a	5.02 ± 3.78 ab	127 ± 60.7	51.70 ± 38.5	49.51	914 ± 301
**Upper basal roots**	**Ctl**	14.0 ± 8.13	**0.00 b**	9.37 ± 4.7	42.9 ± 28.1	56.9 ± 25.6	5.63 ± 4.86	**7.00 ± 6.12 a**	0.47 ± 0.74 ab	28.0 ± 43.9	2.47 ± 4.69	25.9 ± 20.5	10.80 ± 4.3	15.40	219 ± 138
	**Tag**	20.3 ± 16.0	2.25 ± 2.52 ab	4.07 ± 3.4	31.5 ± 21.3	50.3 ± 33.5	5.12 ± 4.24	**0.00 b**	0.00 b	14.6 ± 35.8	0.80 ± 1.43	23.2 ± 29.8	16.37 ± 21.8	13.73	182 ± 142
	**CCM**	33.5 ± 23.3	**6.22 ± 5.51 a**	15.64 ± 5.9	78.7 ± 60.3	64.6 ± 39.0	14.0 ± 7.69	11.1 ± 6.70 a	1.58 ± 0.97 a	12.8 ± 22.5	0.51 ± 1.25	32.9 ± 26.2	13.64 ± 11.6	21.39	307 ± 180
	**BB**	19.7 ± 22.7	1.92 ± 3.49 ab	13.64 ± 11.7	58.6 ± 50.7	61.5 ± 27.6	8.97 ± 6.99	4.90 ± 6.15 ab	0.77 ± 0.92 ab	16.5 ± 32.6	1.82 ± 2.23	42.0 ± 37.9	13.94 ± 8.7	22.81	267 ± 170
	**AM**	10.3 ± 8.82	1.11 ± 2.71 ab	16.55 ± 7.9	49.9 ± 42.0	60.4 ± 31.6	5.98 ± 8.66	8.92 ± 7.03 a	0.67 ± 0.74 ab	36.5 ± 41.3	1.38 ± 1.60	36.1 ± 19.8	16.14 ± 9.6	20.95	265 ± 109
	**SynC**	12.8 ± 8.89	1.00 ± 1.65 ab	16.66 ± 8.7	44.7 ± 23.2	51.9 ± 20.4	6.01 ± 4.18	**0.96 ± 1.51 b**	0.16 ± 0.40 b	10.9 ± 26.8	0.44 ± 1.08	27.0 ± 17.4	13.46 ± 4.1	10.36	196 ± 78

**Figure 2.**
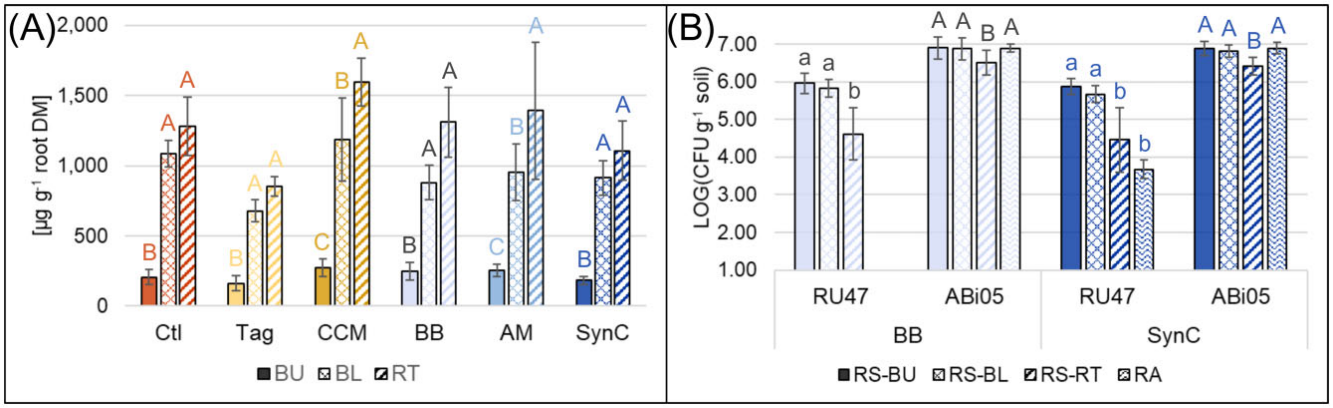
(A) Concentration of total phytoalexins in three parts of the root system (BU: upper basal root; BL: lower basal root; and RT: root tip) of apple M.26 after growth in ARD-affected soil for 42 days under different treatments: Ctl: untreated ARD soil; Tag: amendment of *T. patula*; CCM: amendment of a catch crop mixture; BB: inoculation of beneficial bacteria; AM: inoculation of arbuscular mycorrhiza; and SynC: inoculation of a synthetic community comprising BB and AM. Different letters indicate significant differences between root parts, tested separately per treatment according to ANOVA and *post hoc* Tukey’s HSD test (*n* = 6, *p* < 0.05). (B) CFU counts of inoculated bacteria *Pseudomonas* sp. RU47 and *B. atrophaeus* ABi05 in the rhizosphere (RS) sampled from different root parts or in root-affected soil (RA) in treatments BB and SynC. Different letters (lowercase letters for RU47 and capital letters for ABi05) indicate significant differences between RS root parts or RA according to ANOVA and *post hoc* Tukey’s HSD test (*n* = 12, *p* < 0.05, detection limit <1 × 10^2^ CFU g^−1^).

When comparing the total phytoalexin concentrations among the root parts, we observed the highest concentration in the root tips (>900 µg g^−1^ DM) in all treatments (Fig. 2A). Compared to the root tips, total phytoalexin concentrations were moderately lower in the lower basal roots in all treatments, being significant only for the treatments CCM and AM. In the upper basal roots, total phytoalexin concentrations were overall low (<300 µg g^−1^ DM) and significantly lower compared to the lower basal roots and root tips for all treatments.

### CFU counts of bacterial inoculants were higher in rhizospheres of upper basal roots compared to root tips

CFU of RU47 and ABi05 in the rhizosphere from three root parts (upper and lower basal roots, root tips) and in root-affected soil of treatments BB and SynC were detected after seven weeks, indicating their successful co-colonization (Fig. 2B). No CFUs were detected in the respective samples of the Ctl. In the rhizospheres of upper and lower basal roots, RU47 was detected with ~10^6^ CFU g^−1^ soil and ABi05 with ~10^7^ CFU g^−1^ soil. Interestingly, CFU counts of RU47 and ABi05 were significantly lower in the rhizosphere from root tips compared to the rhizosphere from upper and lower basal roots in treatments BB and SynC. In treatment BB, no CFU counts of RU47 were detected in the root-affected soil, while it was detected with 7 × 10^3^ CFU g^−1^ soil in SynC. Comparing BB and SynC, CFU counts of the bacterial inoculants were similar between the two treatments, except that RU47 was detected in the root-affected soil of SynC. Comparing ABi05 and RU47 CFU counts, RU47 CFUs were at least one order of magnitude lower than ABi05 CFUs, and spatially decreasing CFUs along the root were mainly observed for RU47. Heat shock treatment and selective plating revealed that the majority of ABi05 cells were present in their vegetative form (~10^6^ CFU g^−1^ soil), and only a small proportion was present as spores (~10^4^ CFU g^−1^ soil) in both treatments, BB and SynC (Table S2).

### Catch crop amendments and microbial inoculants shaped the microbial diversity in rhizosphere and root-affected soil

Compared to Ctl samples, bacterial/archaeal alpha-diversity of the treatments changed stronger in rhizosphere, closer to the plant, than in root-affected soil (Fig. S4). In the rhizosphere, the indices Shannon (diversity), Chao1 (richness), and Pielou (evenness) were lower for treatments BB and AM compared to Ctl. Interestingly, the Shannon index was significantly higher in the SynC treatment than in BB or AM. In root-affected soil, Chao1 remained unchanged across treatments, but Tag exhibited the highest Shannon index. Compared to the Ctl, CCM and AM had significantly lower Shannon and Pielou indices. Changes in fungal alpha-diversity were mainly observed in root-affected soil, while in rhizosphere, no differences between the Ctl and any treatment were observed (Fig. S4). The fungal Chao1 index was significantly lower in root-affected soil of all treatments compared to the Ctl. Pielou and Shannon indices were lower in root-affected soils of treatments Tag and CCM compared to Ctl.

**Figure 3.**
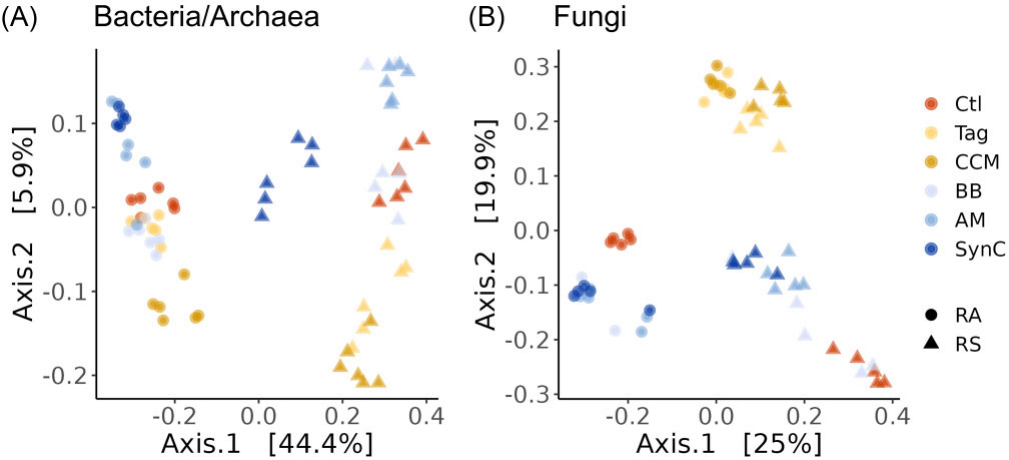
PCoA using a Bray–Curtis dissimilarity matrix based on rarefied square root transformed count data of bacterial/archaeal (A) and fungal (B) communities in the rhizosphere (RS) or root-affected soil (RA) of apple M.26 after growth in ARD-affected soil for 42 days under different treatments: Ctl: untreated ARD soil; Tag: amendment of *T. patula*; CCM: amendment of a catch crop mixture; BB: inoculation of beneficial bacteria; AM: inoculation of arbuscular mycorrhiza; and SynC: inoculation of a synthetic community comprising BB and AM.

Regarding bacterial/archaeal beta-diversity (quantification of species composition using Bray–Curtis distances), the factors microhabitat (rhizosphere or root-affected soil) and treatment strongly influenced these communities, explaining 40.8% and 13.4% of dissimilarity (Fig. 3A, Table 3), respectively. Also, microhabitat-treatment interaction shaped the bacterial/archaeal beta-diversity, explaining 9.3% of dissimilarity. For fungi, the effect of microhabitat was notably lower compared to bacteria/archaea, explaining 18.0% of dissimilarity between samples (Fig. 3B, Table 3). Here, the treatment was the strongest factor driving fungal beta-diversity, explaining 26.4% of dissimilarity. As observed for bacterial/archaeal communities, a strong effect of microhabitat-treatment interaction on beta-diversity was observed. Interestingly, PCoA indicated a low fungal dissimilarity between microhabitats for treatments Tag and CCM, while microhabitats on the *x*-axis separated samples of the other treatments (Fig. 3B). Pairwise comparisons of individual treatments to the Ctl revealed that bacterial/archaeal beta-diversity in rhizosphere differed significantly between each treatment and the Ctl, and in root-affected soil between Tag, CCM, or SynC and Ctl (Table S3). Performing pairwise PERMANOVA on fungal communities revealed significant dissimilarities between Ctl and Tag, CCM, or AM in rhizosphere and between Ctl and Tag in root-affected soil (Table S3).

**Table 3.** Permutational analysis on variance (PERMANOVA) on square root transformed count data assessing the significance of microhabitat (rhizosphere and root-affected soil) and treatment (Ctl: untreated ARD soil; Tag: amendment of *T. patula*; CCM: amendment of a catch crop mixture; BB: inoculation of beneficial bacteria; AM: inoculation of arbuscular mycorrhiza; and SynC: inoculation of a synthetic community comprising BB and AM) on bacterial/archaeal and fungal beta-diversity.

	Bacteria/Archaea				Fungi			
	Df	*R* ^2^	*F*	*p*	Df	*R* ^2^	*F*	*p*
Microhabitat (mh)	1	0.408	66.08	<0.001	1	0.180	23.61	<0.001
Treatment (treat)	5	0.134	4.33	<0.001	5	0.264	6.92	<0.001
mh:treat	5	0.093	3.02	<0.001	5	0.189	4.95	<0.001
Residual	59	0.36			48	0.367		

### Catch crop amendments and microbial inoculants modulated the bacterial/archaeal community composition in rhizosphere and root-affected soil

**Figure 4.**
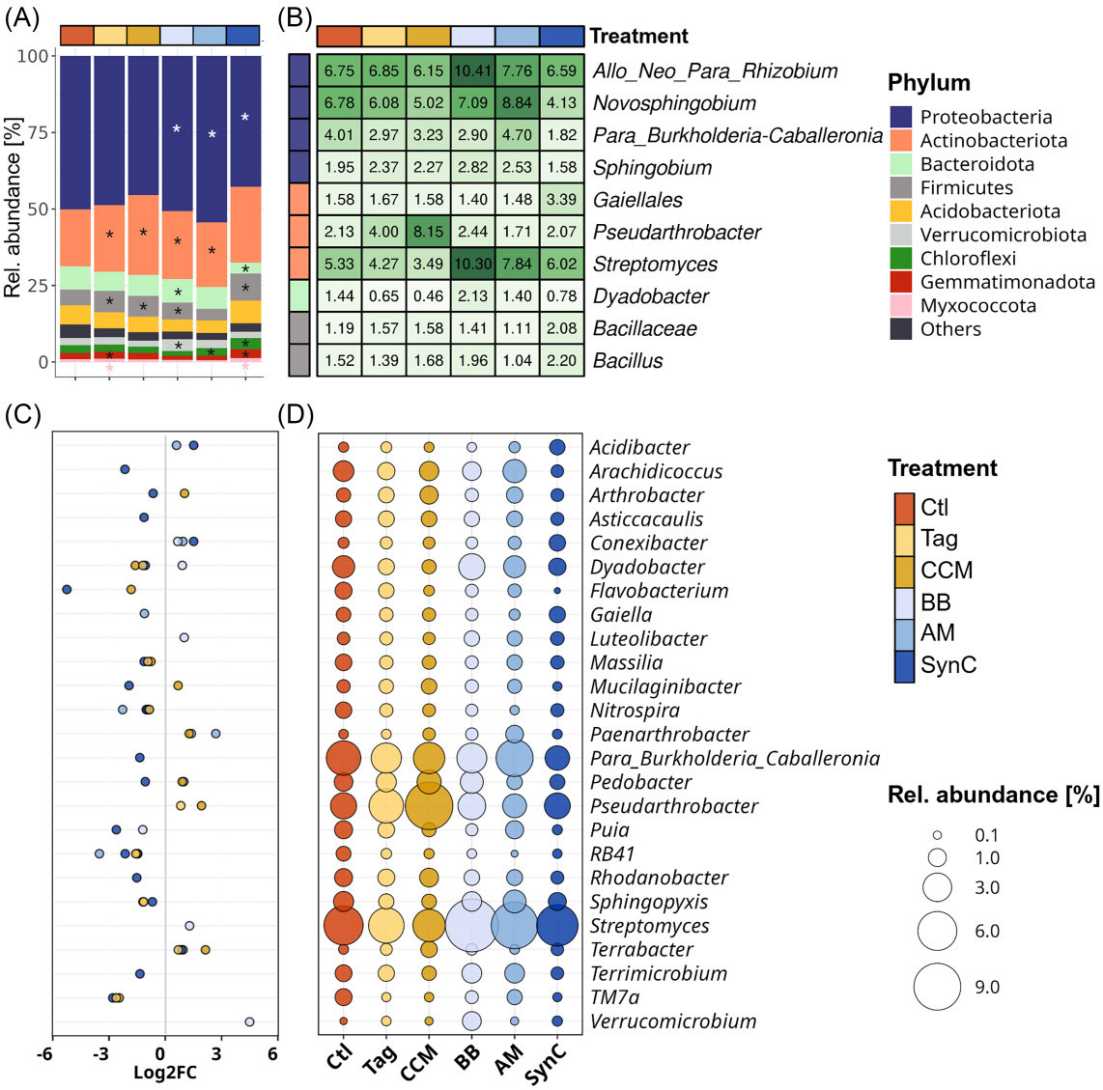
Bacterial/archaeal community composition in the rhizosphere of M.26 grown for 42 days in ARD soil under different treatments (*n* = 6): Ctl: untreated ARD soil; Tag: amendment of *T. patula*; CCM: amendment of a catch crop mixture; BB: inoculation of beneficial bacteria; AM: inoculation of arbuscular mycorrhiza; and SynC: inoculation of a synthetic community comprising BB and AM. Average relative abundance (rel. abundance) of bacterial phyla in the different treatments (A). Asterisks indicate significant differential abundance compared to the Ctl. Average rel. abundance of the ten dominant bacterial taxa across treatments (B). Values in each cell represent average rel. abundances. Log2 fold-change (Log2FC) values (C) and average rel. abundance (D) of differentially abundant taxa between Ctl and treatments Tag, CCM, BB, AM, or SynC. Negative Log2FC values indicate lower abundance and positive Log2FC values indicate higher abundance compared to the Ctl. Dot sizes represent average rel. abundances. Differential abundance testing was done using negative binomial distribution using DeSeq2. Taxa were considered differentially abundant for *p-adj* < 0.05 (Benjamini–Hochberg correction). Post-analysis, taxa were filtered for RA > 0.5% and Log2FC <-1/>+1. Exact values of relative abundances and Log2FC values are provided in Tables S3 and S4.

The dominant bacterial phyla in the rhizosphere were Proteobacteria, Actinobacteriota, Bacteroidota, Firmicutes, and Acidobacteriota. Notable differences between Ctl and treatments were already evident at the phylum level (Fig. 4A). Compared to the Ctl, the relative abundance of Actinobacteriota was significantly higher in rhizospheres of Tag, CCM, BB, and AM and of Firmicutes in Tag, CCM, BB, and SynC (Fig. 4A, Table S4). Interestingly, the strongest differences at the phylum level were observed for treatment SynC compared to the Ctl, with Proteobacteria and Bacteroidota being significantly lower and Firmicutes, Chloroflexi, Gemmatimonadota and Myxococcota being significantly higher in SynC. Distinct patterns between the Ctl and treatments in the rhizosphere were also observed among the ten most abundant bacterial taxa (Fig. 4B). The dominant genera were common rhizosphere colonizers such as *Allo_Neo_Para_Rhizobium, Novosphingobium, Pseudarthrobacter*, and *Streptomyces*. We identified several differentially abundant bacterial genera between the Ctl and the different treatments, indicating a microbiome modulation by catch crop amendments and microbial inoculations (Fig. 4C and D, Table S5). Treatment SynC had the highest number of differentially abundant genera compared to the Ctl (Table S5). The relative abundance of RB41, possibly assigned to Acidobacteriota, was significantly lower for all treatments, while *Terrabacter* was significantly higher in all treatments except AM, compared to the Ctl. The relative abundance of *Pseudarthrobacter* was significantly higher in the catch crop amended treatments Tag (4.0%) or CCM (8.2%) than in Ctl (2.1%). Relative abundance of TM7a was significantly lower in treatments Tag, CCM, and SynC compared to Ctl. The relative abundance of *Dyadobacter* was significantly lower in treatments Tag (0.7%), CCM (0.5%), and SynC (0.8%), while in treatment BB, its relative abundance was higher (2.1%) than in Ctl (1.4%). The relative abundance of *Streptomyces* was higher in all inoculation treatments (BB: 10.3%, AM: 7.8%, SynC: 6.0%) compared to 5.3% in the Ctl, but the difference was significant only in BB. *Para_Burkholderia–Caballeronia* was significantly lower exclusively in the rhizosphere of treatment SynC (1.8%) compared to Ctl (4.0%). Although *Bacillus* was among the dominant taxa in the rhizosphere, it was not differentially abundant between the Ctl and any of the five treatments, including BB and SynC, where ABi05 and RU47 were inoculated. We identified ASV26 and ASV135 having 100% sequence identity with *B. atrophaeus* type strains. Although generally low, their relative abundance was higher in BB (ASV26: 0.37%; ASV135: 0.12%) and SynC (ASV26: 0.20%; ASV135: 0.03%) compared to the Ctl (ASV26: 0.003%; ASV135: 0.0%). Compared to the Ctl (0.5%), *Pseudomonas'* relative abundance was significantly higher in BB (0.7%), with ASV422 showing 100% sequence identity to the inoculant strain RU47, but not in SynC (0.5%).

The dominant bacterial phyla in root-affected soil were Proteobacteria, Actinobacteriota, Firmicutes, and Acidobacteriota (Fig. S5A). Treatments CCM, AM, and SynC resulted in clear shifts at the phylum level compared to Ctl, while for Tag and BB only a few changes were observed. The relative abundance of Firmicutes was significantly higher in all treatments except Tag compared to Ctl (Fig. S5A, Table S4). The archaeal phylum Crenarchaeota was significantly lower in root-affected soil with catch crop amendments. At the same time, the inoculation with AM or SynC led to a significantly higher relative abundance of this phylum compared to Ctl. Among the dominant taxa in root-affected soil were unclassified Gaiellales, *Pseudarthrobacter, Bacillus*, and unclassified Bacillaceae (Fig. S5B; Table S6). Compared to the rhizosphere, fewer taxa with differential abundance between the treatments and Ctl were observed, with treatment SynC showing the highest number of differentially abundant taxa compared to Ctl (Fig. S5C and D). *Bacillus* was significantly higher in all inoculation treatments, compared to Ctl (3.6%), and was highest in treatments BB (6.1%) and SynC (7.3%), which received *B. atrophaeus* ABi05 as inoculant (Fig. S5C and D; Table S6). As observed in the rhizosphere, we identified ASV26 and ASV135 in notably higher relative abundance in BB (ASV26: 1.21%; ASV135: 0.39%) and SynC (ASV26: 2.12%; ASV135: 0.72%) compared to the Ctl (ASV26: 0.06%; ASV135: 0.006%). *Pseudomonas* relative abundance was low in root-affected soils of all treatments, and we did not identify any ASV to be identical to the RU47 genome sequence. Notably, *Ligilactobacillus* was detected only in AM- and SynC-treated root-affected soils and was absent in the other treatments and Ctl. Relative abundances of *Candidatus* Nitrocosmicus and *Candidatus* Nitrosotalea were lower in catch crop treatments and higher in inoculation treatments AM and SynC compared to Ctl.

### Catch crop amendments modulated the fungal community composition in rhizosphere and root-affected soil

**Figure 5.**
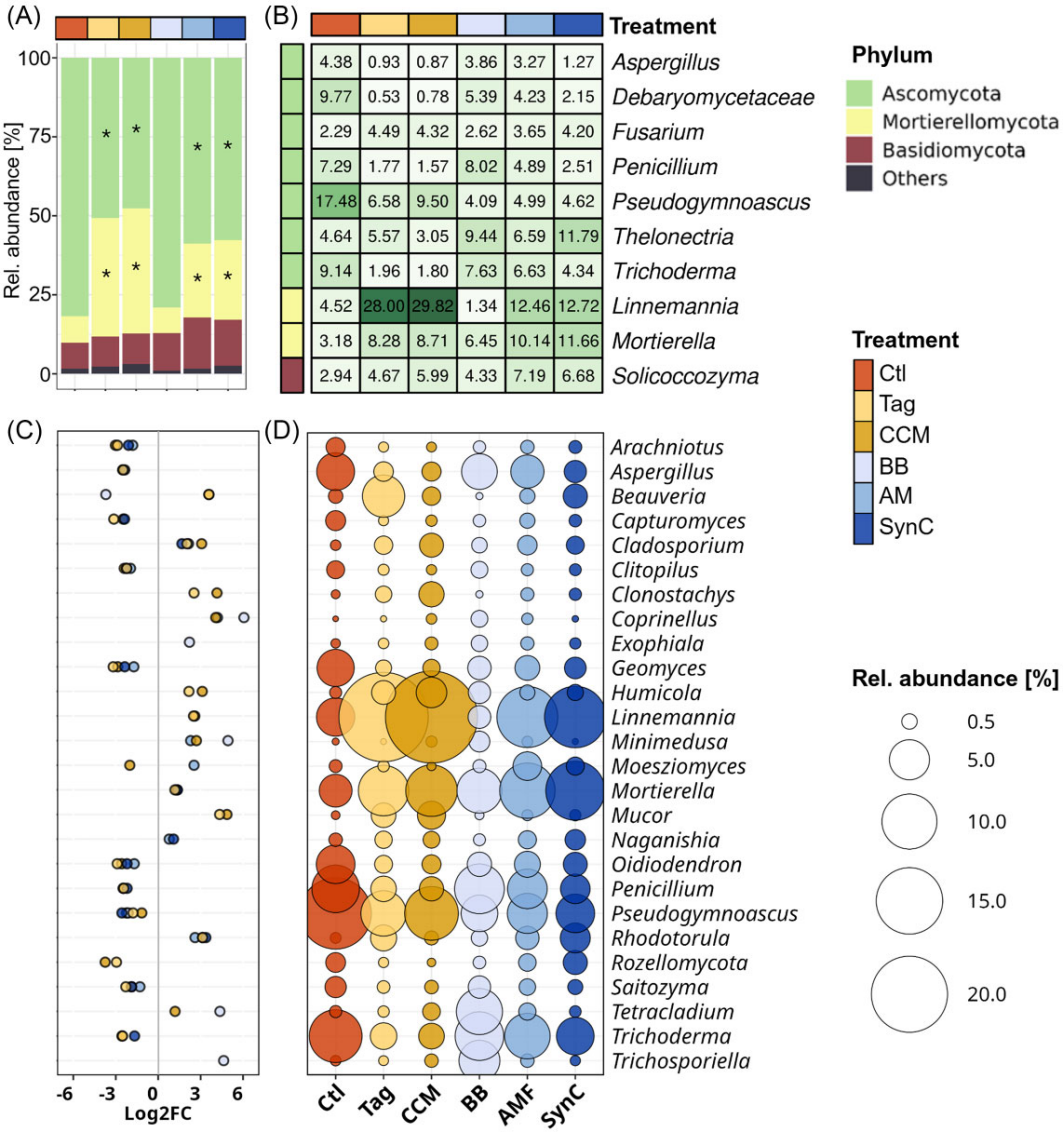
Fungal community composition in the rhizosphere of M.26 grown for 42 days in ARD soil under different treatments (*n* = 5): Ctl: untreated ARD soil; Tag: amendment of *T. patula*; CCM: amendment of a catch crop mixture; BB: inoculation of beneficial bacteria; AM: inoculation of arbuscular mycorrhiza; and SynC: inoculation of a synthetic community comprising BB and AM. Average relative abundance (rel. abundance) of bacterial phyla in the different treatments (A). Asterisks indicate significant differential abundance compared to the Ctl. Average rel. abundance of the ten dominant bacterial taxa across treatments (B). Values in each cell represent average rel. abundances. Log2 fold-change (Log2FC) values (C) and average rel. abundance (D) of differentially abundant taxa between Ctl and treatments Tag, CCM, BB, AM, or SynC. Negative Log2FC values indicate lower abundance and positive Log2FC values indicate higher abundance compared to the Ctl. Dot sizes represent average rel. abundances. Differential abundance testing was done using negative binomial distribution using DeSeq2. Taxa were considered differentially abundant for *p-adj* < 0.05 (Benjamini–Hochberg correction). Post-analysis, taxa were filtered for RA > 0.5% and Log2FC <-1/>+1. Exact values of relative abundances and Log2FC values are provided in Tables S6 and S7.

The dominant fungal phyla in the rhizosphere were Ascomycota, Mortierellomycota, and Basidiomycota. We observed differences in relative abundance between Ctl and all treatments except BB (Fig. 5A). Relative abundance of Ascomycota was significantly lower in rhizospheres of Tag, CCM, AM, and SynC. At the same time, Mortierellomycota were significantly more abundant in these treatments compared to Ctl (Fig. 5A, Table S7). Most dominant fungal taxa in the rhizosphere were also observed to be differentially abundant between the Ctl and different treatments (Fig. 5B–D, Table S8). Also, we observed the highest number of differentially abundant taxa in the rhizospheres of catch crop treatments, while fewer taxa were identified for the inoculation treatments (Table S8). With 17.5% relative abundance, *Pseudogymnoascus* was the dominant taxon in Ctl, while it was significantly lower in rhizospheres of all treatments (Tag: 6.6%; CCM: 9.5%; BB: 4.1%; AM: 5.0%; SynC: 4.6%) (Fig. 5B–D). *Oidodendron* and *Geomyces* had significantly higher relative abundances in Ctl compared to all treatments except BB (Fig. 5C and D, Table S8). In contrast, the relative abundance of *Rhodotorula* was significantly lower in Ctl than in all treatments except BB. Relative abundances of *Aspergillus, Penicillium*, and *Trichoderma* were significantly higher in Ctl rhizospheres than in Tag, CCM, or SynC (Fig. 5B–D, Table S8). In treatments Tag and CCM, the dominant taxa were *Linnemannia* (Tag: 28.0%, CCM: 29.8%) and *Mortierella* (Tag: 8.3%, CCM: 8.7%), which are members of the phylum Mortierellomycota, whose relative abundance was significantly lower in Ctl (*Linnemannia*: 4.5%, *Mortierella*: 3.2%). Also in AM and SynC rhizospheres, the *Mortierella* relative abundances were significantly higher than in Ctl.

Like in rhizosphere, the dominant fungal phyla in root-affected soil were Ascomycota, Mortierellomycota, Basidiomycota, and Mucoromycota (Fig. S6A). Mortierellomycota were significantly more abundant in Tag and CCM and lower in BB and AM compared to Ctl (Table S7). The relative abundances of Mucoromycota were significantly higher in all treatments, except SynC, compared to Ctl. Treatment-dependent differences among the dominant taxa were less pronounced in root-affected soil than in the rhizosphere (Fig. S6B). Still, differential abundance analysis revealed several taxa that differed in relative abundance between the treatments and Ctl (Fig. S6C and D, Table S9). In all treatments, the relative abundance of *Zygotorulaspora* was significantly lower (0.02%–0.4%) compared to 0.9% in Ctl. The relative abundance of *Mucor* was significantly higher in Tag (1.6%), CCM (1.8%), and AM (4.2%) compared to 0.2% in Ctl. Like in the rhizosphere, the *Linnemannia* relative abundance was strikingly higher in Tag (22.6%) and CCM (28.4%) compared to 9.9% in Ctl. In contrast, its relative abundance was <3% in all inoculation treatments and significantly lower than in Ctl. Exclusively in root-affected soil of SynC, *Corpinellus* was significantly high abundant (14.0%), while very low in the Ctl (0.2%). The AM inoculants *R. irregulare* and *F. mosseae* were not among the dominant fungal taxa and no ASVs representative of the inoculated AM were detected. In all treatments, the relative abundance of Glomeromycota was low in the rhizosphere (<0.05%) and in root-affected soil (<0.1%), and no differentially abundant ASVs or taxa of this phylum between AM or SynC compared to the Ctl were identified.

## Discussion

This study assessed the potential of catch crop amendments and microbial inoculants to attenuate negative plant–soil feedback under apple replanting. In the following, we will discuss both approaches with regard to alterations in soil nutrients, changes in the biochemical plant response, and the modulation of the rhizosphere and root-affected soil microbiomes.

### Potential of catch crop amendments to improve soil health and plant performance

The improved plant performance that we partially observed in the catch crop treatments can partly be explained by increased soil fertility, i.e. elevated levels of N_min_, Na, Zn, K (Tag), Mg (Tag), and Mn (CCM) (Table 1). The incorporation of the chopped plant debris loosened the soil and might have provided additional niches for the colonization of microbes as different plants harbor diverse microbiomes and contribute to the soil metabolic activity (File S1, Table S1) (Reinhold-Hurek et al. 2015, Bashir et al. 2021). The alteration of soil microbiome functions through root metabolites of catch crops depends on their composition. Several studies reported beneficial effects of catch crop amendments on the soil microbiome compared to fallow soil (Strickland et al. 2019, Li et al. 2022, Seitz et al. 2024). Gentsch et al. (2020) showed a positive association between plant species diversification and microbial biomass, indicating the benefits of a crop mixture compared to a single catch crop. Our results support this finding and showed that in CCM, and to a lesser extent in Tag, the soil microbial activity at the experiment set-up was significantly higher compared to the Ctl (File S1, Table S1). Positive effects on apple plant growth of catch cropping with *Tagetes* in ARD soils were shown previously (Yim et al. 2017, Kanfra et al. 2021). Species diversification in CCM could be advantageous compared to the single species amendment Tag. The highest root length in CCM could be a consequence of the high soil microbial activity due to the input of organic matter, providing diverse nutrient sources for microbes and ultimately resulting in better soil fertility.

We assume that the observed plant growth promotion in the catch crop treatments was a result of combined mechanisms. However, since the apple plants in the greenhouse experiment were fertilized with a slow-release fertilizer, the macronutrients were likely sufficient in all treatments and catch crop effects might rather be changes in plant-available micronutrients. The nutrient status of both, soils at planting as well as plants at the end of the experiment, should be taken into consideration in future research. In addition to the input of nutrients, improved nutrient retention and enhancements in soil structure likely were at play in the CCM and Tag amended soils. Decomposed catch crop biomass likely contributed immediately to the nutrient pool through mineralization, while at the same time stimulating microbial turnover through increased organic matter contents (Koch et al. 2020, Gentsch et al. 2020). Compared to the single catch crop Tag, the diverse plant species mixture in CCM may have mobilized a broader spectrum of nutrients through varying root exudates and architectures, facilitating enhanced nutrient cycling and mitigating nutrient loss (Wendling et al. 2015, Heuermann et al. 2022). Moreover, catch crop amendments were shown to provide increased microbial biomass and rhizodeposition, resulting in improved soil aggregation, improved pore structure, and nutrient retention (Ranaldo et al. 2020, Gentsch et al. 2024). Taken together, nutrient addition, activation of soil microbiota, and improvement of the soil structure are likely complementary mechanisms, which in their combination could explain the increased fertility and partially improved plant growth observed in the CCM and Tag treatments.

Although Tag and CCM significantly increased several soil nutrients and had the strongest positive effect on root growth, apple plants responded differently to the two catch crop amendments in terms of root phytoalexins by producing different compounds and concentrations. Plants synthesize phytoalexins in response to biotic or abiotic stress as part of their defense system. Phytoalexins are diverse low molecular weight compounds with antimicrobial activity, which allow plants to control the invasion by unwanted microbes (Piasecka et al. 2015, Kaur et al. 2022). Apple and other members of the Malinae produce biphenyls and dibenzofurans (Busnena et al. 2023). Certain phytoalexins, including 3-hydroxy-5-methoxybiphenyl, noreriobofuran, and noraucuparin, were previously shown to accumulate in roots grown in ARD-affected soils compared to disinfected soil or soil without replanting history (Balbín-Suárez et al. 2021, Siefen et al. 2024). Also, the exudation rates of phytoalexins into the soil differ for individual compounds, indicating that their exudation is a controlled process (Busnena et al. 2021). The direct influence of exuded phytoalexins on the rhizosphere microbiome, as demonstrated by differential inhibitory activities (Busnena et al. 2025), highlights the strong plant–soil feedback under replanting conditions. Because total phytoalexin concentrations in Tag and CCM did not differ from the Ctl, we analyzed the individual compounds in the three root parts separately, which revealed few significant differences. In Tag, certain phytoalexin compounds were reduced compared to the Ctl, i.e. noreriobofuran, which was one of the dominant compounds in the Ctl, was absent from roots grown in Tag. Catch cropping with *Tagetes* is commonly used to reduce populations of plant–parasitic nematodes, mediated through the production of thiophenes (Hooks et al. 2010). Our data suggest that the metabolites provided in Tag extenuated the response of the apple plants resulting in slightly improved plant growth (Table 2, Fig. S3). The opposite was observed for CCM, in which phytoalexin levels were generally high, along with improved plant performance. In roots of apple plants grown in CCM, the concentration of noraucuparin, 3-hydroxy-4,5-demethoxybiphenyl, and hydroxyeriobofuran isomer 1 were elevated compared to Tag or Ctl (Table 2), suggesting that they were produced in response to CCM. The CCM comprised twelve plant species, mainly *Brassicaceae*, legumes, and *Tagetes. Brassicaceae* are rich in glucosinolates that can be hydrolyzed into isothiocyanates when chopped and incorporated into the soil (Hanschen and Winkelmann 2020). Possibly, the CCM activated biofumigation effects that might have induced the suppression of potential plant pathogens and triggered phytoalexin production. A similar mechanism was described for glucosinolate degradation products in *Arabidopsis thaliana* (Andersson et al. 2015). However, we did not observe reduced relative abundances of potential plant pathogens in CCM (Figs 4 and 5). We instead assume that the amendment of CCM induced shifts in the microbiome toward the enrichment of copiotrophic microbes and increased the soil microbial activity (Table S1). Presumably, their high density was sensed by the plant, which likely resulted in elevated phytoalexin levels, indicating that apple plants do not differentiate between beneficial, commensal, or pathogenic microbes in their phytoalexin response. Similarly, phytoalexins do not selectively inhibit detrimental microbes (Busnena et al. 2025). The different phytoalexin patterns triggered by Tag and CCM amendments suggest that a deeper understanding of the underlying mechanisms of the plant–microbe–plant interaction (catch crop–microbiome–apple) is needed to predict their efficacy. So far, the interplay between catch crop amendments, phytoalexins and the role of the microbiome has not been studied well and should be addressed in the future.

Catch crops primarily modulated fungal communities, likely contributed to soil structural and nutrient changes and attracted destruents. The pronounced impact of catch crop amendments on fungal communities aligns with studies highlighting the role of organic inputs in shaping fungal diversity and function (Strickland et al. 2019, Li et al. 2022, Seitz et al. 2024). Incorporating plant debris likely provided additional ecological niches, promoting saprotrophic fungi such as *Linnemannia* and *Mortierella*, known for organic matter decomposition and plant growth promotion (Debode et al. 2016, Zhang et al. 2020, De Tender et al. 2024). The significantly lower relative abundance of *Trichoderma* in the rhizosphere of Tag and CCM might be due to antagonistic effects between *Trichoderma* and *Linnemannia* or *Mortierella* that were highly enriched in those treatments compared to the Ctl. Besides an antagonistic direct effect, the root metabolites of the catch crops could have induced antifungal effects in Tag or CCM. Li et al. (2023) reported that *Trichoderma* and *Linnemannia* were inhibited by switchgrass root extracts and highlighted that compounds that inhibited *Trichoderma* were distinct from compounds that inhibited *Linnemannia*. Like *Trichoderma, Pseudogymnoascus* and *Geomyces* were abundant in Ctl soils and significantly decreased in relative abundance in inoculated soils. The dominance of *Pseudogymnoascus* in the Ctl supports previous findings linking their dominance to ARD-affected soils (Hauschild et al. 2024). Their accumulation could indicate microbiome disturbances related to replanting stress. The depletion of TM7a in Tag and CCM could result from altered root exudation or microbial networks. TM7a, a member of *Saccharimonadaceae*, relies on co-metabolism with other microbes and is associated with organic matter degradation (Kublik et al. 2022, Wang et al. 2024b). Its reduction suggests a restructuring of microbial consortia, favoring functionally beneficial taxa. Catch crop amendments also influenced archaeal communities, depleting ammonia-oxidizing archaea (AOA) such as *Candidatus* Nitrocosmicus and *Candidatus* Nitrosotalea. AOA thrive in nutrient-poor agricultural soils, particularly under conventional management (Wilhelm et al. 2023). Their reduced abundance suggests catch crop amendments to improve nutrient availability, reducing the competitive advantage of AOA and enhancing soil health.

### Microbial inoculations induced microbiome modulations but had minor effects on plant response, soil nutrients, and plant growth

None of the tested microbial inoculations improved plant performance under the tested conditions, leading us to reject our hypothesis for treatments BB, AM, and SynC. In our study, the apple plants were not set under abiotic stress like nutrient or water limitations and were grown under controlled greenhouse conditions. Presumably, the plant-beneficial effect of the inoculants would have been activated when the plants suffered from such limitations or faced changing environmental conditions (Eltlbany et al. 2019, Hett et al. 2023). Based on the detected CFUs seven weeks after inoculant application, we can exclude that the missing improvement in plant performance was due to the failing establishment of the bacterial inoculants. We showed rhizosphere competence of RU47 and ABi05 on apple rootstock M.26, in agreement with a previous study that reported rhizosphere competence of RU47 and *B. velezensis* FZB42 on apple for at least four weeks, when applied as single inoculants (Hauschild et al. 2024). Now, we extend this knowledge for RU47 to seven weeks and could show that RU47 and ABi05 co-colonized the rhizosphere of different root parts. Mainly RU47 CFU counts followed a gradient of decreasing CFUs with increasing distance to the initial root system (Fig. 2B). This observation is in line with findings by Yim et al. (2025), who also observed higher inoculant CFUs in the basal roots compared to the root tips in a seed inoculation experiment with maize. The decreasing CFUs along the root were less pronounced for ABi05 than RU47, which might be due to the different inoculation techniques. While RU47 was inoculated via root dipping, meaning that the application of RU47 was limited to the initial root system, ABi05 spores were incorporated into the soil where they were also present during root growth. We further assume that the decreasing CFUs toward the lower basal roots and root tips occurred due to the higher exudation of primary and secondary metabolites in younger roots and root tips, inducing their repellent effect on microbes to protect the vulnerable root tissue (Hinsinger et al. 2005, Proctor and He 2017). This is supported by our data showing that, with decreasing CFUs, increasing root phytoalexin concentrations were observed along the different root parts (Fig. 2). Siefen et al. (2024) showed higher phytoalexin levels in apple roots grown in ARD-affected soils after four compared to eight weeks. Our data support their finding and suggest that their observation could be explained by sampling younger roots (root tips and lower basal roots) at early time points. In comparison, the proportion of older roots (upper basal roots) with lower phytoalexin concentrations would increase over time. The apparent differences in phytoalexin concentrations in the different root parts suggest that the root sampling procedure and plant age are important factors that should be considered in future samplings. It is well known that roots contain different concentrations of phytoalexins in different root zones, with phytoalexins accumulating mainly in actively growing root zones, namely the root tips. Young and elongating root cells depend on an enhanced immunity such as the accumulation of phytoalexins because they are not yet fully developed and still lack a protective epidermis layer (Chuberre et al. 2018). A recent study has shown that phytoalexin production occurred not only in response to pathogen invasion, but also following inoculation with beneficial microbes (Hauschild et al. 2024). This suggests that increased phytoalexin synthesis may be a general response to microbiome disturbance, whether triggered by pathogenic microbes or the introduction of beneficial microbes at high cell densities. However, our data did not reveal any differences when comparing concentrations of total phytoalexins and individual compounds between the inoculation treatments and the Ctl (Table 2). Thus, we conclude that the inoculation of beneficial microbes did neither foster a stronger nor induce a weaker plant response than in the already disturbed Ctl.

The detection of RU47 cells in root-affected soil in SynC, but not in BB, indicates that the bacterial inoculants synergistically interact with the AM, which may facilitate the colonization of root-affected soil by RU47 through “hyphal highways” (Warmink et al. 2011, Jiang et al. 2021). Several rhizosphere bacteria can solubilize phosphorus from soil and make it available to the AM and the plant. Sharma et al. (2020b) showed that the co-inoculation of *R. irregulare* and phosphate solubilizing *Pseudomonas* led to significantly higher tomato plant growth compared to their single inoculation. Additive effects of the SynC members could occur due to their complementary beneficial functions or niches that they colonize. Several studies showed that using a consortium of two or more microorganisms can have a stronger modulation effect on the microbiome or improve plant growth better than a single inoculum (Berendsen et al. 2018, Hafiz et al. 2022, Gonҫalves et al. 2023). We observed a significant increase in bacterial/archaeal alpha-diversity in the rhizosphere of SynC compared to BB or AM (Fig. S4), indicating that the combined inoculation of SynC activated their synergistic effects that were not achieved through inoculation of BB or AM alone. Regarding the composition, we observed greater differences between the bacterial/archaeal communities of Ctl and SynC than between the Ctl and BB or AM (Fig. 3A, Table S2). Further, as observed and discussed for Tag and CCM, differential abundance of *Trichoderma* and TM7a was observed in the rhizosphere only after inoculation of SynC, but not in BB or AM (Fig. 4). The postulated synergy between BB and AM when combined into SynC was further supported by the observation that the root length of apple plants was higher in SynC compared to BB or AM (Fig. S3).

The response of bacterial/archaeal communities to microbial inoculation suggests that these treatments influenced microbial interactions with native populations rather than directly improving soil nutrients. The increase in *Streptomyces* in all inoculation treatments suggests stimulation of taxa with potential biocontrol and plant growth-promoting properties (Ding et al. 2010). *Streptomyces* species degrade phenolic compounds and mitigate soil stress, which is relevant in ARD-affected soils where phenolics from previous apple plantations can accumulate (Uroz et al. 2019, Jiang et al. 2022). Similarly, *Bacillus* was enriched in root-affected soil in all inoculation treatments, reinforcing its role in modulating soil microbiomes through competitive interactions and metabolite production (Jacoby et al. 2017). However, we did not find ABi05-related ASVs exclusively in treatments BB or SynC that were inoculated with ABi05, indicating that their differential abundance is not only due to the high densities of the inoculated strain, but that the inoculations promoted native *Bacillus* strains. Regarding the AM inoculated samples, no members of Glomeromycota were found among the differentially abundant fungi in treatments AM or SynC. This is likely due to an already described limitation of ITS2 region in detecting AM. The ITS2 region is poorly suited to amplify Glomeromycota due to primer biases and underrepresentation of AM in reference databases. Although the sequencing data are based on short-read amplicon sequencing of the ITS2 fragment, previous *in silico* assessments have shown that commonly used ITS2 primers perform poorly in capturing AM diversity (Tedersoo et al. 2022, Nilsson et al. 2019). A more targeted approach, such as sequencing longer fragments of the *18S rRNA* or large subunit (*LSU*) *rRNA* genes with AM-specific primers, would likely be more effective for their detection (Morgan and Egerton-Warburton 2017, Kolaříková et al. 2021, Delavaux et al. 2022).

### Catch crops and microbial inoculants cause distinct microbiome modulations

Catch crop amendments and microbial inoculants induced distinct shifts in bacterial, archaeal, and fungal communities, each following a distinct mode of action. Our results suggest that catch crop amendments, particularly diverse mixtures like CCM, offer a promising strategy for microbiome modulation in ARD soils. The amendment of catch crops increased nutrient contents, enriched copiotrophic bacterial and fungal taxa, and likely resulted in higher microbial activity. The enrichment of beneficial fungal taxa, i.e*. Linnemannia* and *Mortierella*, and depletion of potential soil disturbance indicators highlight the potential of catch cropping for restoring a well-functioning microbiome. Microbial inoculants influenced bacterial and archaeal groups but did not increase soil nutrient contents, indicating their indirect influence on the plant. These indirect interactions and direct microbe–microbe interactions might increase plant resilience toward abiotic and biotic stress (Berg et al. 2021). Future studies should explore whether targeted inoculation of beneficial taxa, such as *Linnemannia* or *Mortierella*, could provide a more promising management approach of replant-affected soils compared to the inoculation of BB or AM. While catch crops and microbial inoculants influence microbial communities, their differing modes of action suggest an integrated approach. Our study investigated catch crops and microbial inoculants as separate management strategies, but we did not study their combined application, leaving potentially synergistic effects unexplored. Integrating both strategies could harness complementary mechanisms, combining the structural and nutrient benefits of catch crops with the functional roles of inoculated microbes (Timmusk et al. 2017, Vukicevich et al. 2016). Future studies should explore this interaction to assess whether combined treatments have additive or synergistic effects on plant growth, soil health, and microbiome balance. Combining organic amendments with targeted microbial inoculants may be most effective in restoring soil functioning in replant diseased soils and promoting sustainable apple production.

Despite microbiome modulation, our data did not indicate a dysbiotic rhizosphere community in the Ctl. Evenness indices were comparable across treatments, and no bacterial or fungal taxon dominated the Ctl rhizosphere to suggest pathogenic imbalance. This aligns with findings that ARD is driven by complex plant–soil–microbe interactions rather than a single pathogen (Wang et al. 2022).

## Conclusion

Prospectively, we aim to provide integrated agricultural management strategies that alleviate negative plant–soil feedback under monocropping and contribute to growing healthy plants while maintaining healthy soils. The results presented here indicate that adding catch crops, especially a crop mixture, is a promising strategy that could be easily implemented by practitioners (Dabney et al. 2001). However, it is questionable how the positive impact of a preculture is lingering over the growth period of apple. Therefore, we propose further studies that develop and compare the effects of a preculture to a permanent cover crop during the cultivation period. Regarding the microbial inoculants, positive effects were mainly observed for the inoculation of a synthetic community, comprising BB and AM rather than their separate inoculation.

This study provides insights into the effects of catch crop amendments or microbial inoculants on the microbiome composition in rhizosphere and root-affected soil, but not the microbiome functions. However, increasing evidence suggests that functional rather than taxonomic microbial diversity drives soil health in agroecosystems (Wagg et al. 2014, Manning et al. 2018). Future studies should benefit from RNA-based transcriptomic and cultivation-dependent approaches to better understand the complex plant–soil–microbiome interactions and underlying modes of action under different management strategies.

## Supplementary Material

fiaf055_Supplemental_Files

## Data Availability

Raw sequences derived from *16S rRNA* gene and ITS fragment amplicon sequencing, including metadata, were deposited at the NCBI sequence read archive under the BioProject number PRJNA1227435. All R code used for the bioinformatic analyses is available under https://github.com/krishauschi/Ordiamur_ms3.

## References

[bib1] Abarenkov K, Zirk A, Piirmann T et al. UNITE general FASTA release for fungi. Version 04.04. London: UNITE Community, 2024. 10.15156/BIO/2959332 (21 February 2025, date last accessed).

[bib2] Adesina MF, Grosch R, Lembke A et al. *In vitro* antagonists of *Rhizoctonia solani* tested on lettuce: rhizosphere competence, biocontrol efficiency and rhizosphere microbial community response. FEMS Microbiol Ecol. 2009;69:62–74. 10.1111/j.1574-6941.2009.00685.x. 19486156

[bib3] Alagna F, Balestrini R, Chitarra W et al. Getting ready with the priming: innovative weapons against biotic and abiotic crop enemies in a global changing scenario. In: Priming-Mediated Stress and Cross-Stress Tolerance in Crop Plants. Amsterdam: Elsevier Inc, 2020, 35–56.

[bib4] Andersson MX, Nilsson KA, Johansson ON et al. Involvement of the electrophilic isothiocyanate sulforaphane in *Arabidopsis* local defense responses. Plant Phisiol. 2015;167:251–61. 10.1104/pp.114.251892. PMC4281013 25371552

[bib5] Balbín-Suárez A, Jacquiod S, Rohr A-D et al. Root exposure to apple replant disease soil triggers local defense response and rhizoplane microbiome dysbiosis. FEMS Microbiol Ecol. 2021;97:4. 10.1093/femsec/fiab031. 33587112

[bib6] Banerjee S, van der Heijden MGA. Soil microbiomes and one health. Nat Rev Microbiol. 2023;21:6–20. 10.1038/s41579-022-00779-w. 35999468

[bib7] Bashir O, Ali T, Baba ZA Soil organic matter and its impact on soil properties and nutrient status. In: Dar Bhat (ed.), Microbiota and biofertilizers. Vol. 2, Cham: Springer International Publishing, 2021, 129–59.

[bib8] Behr JH, Kampouris ID, Babin D et al. Beneficial microbial consortium improves winter rye performance by modulating bacterial communities in the rhizosphere and enhancing plant nutrient acquisition. Front Plant Sci. 2023;14:1232288. 10.3389/fpls.2023.1232288. 37711285 PMC10498285

[bib9] Berendsen RL, Vismans G, Yu K et al. Disease-induced assemblage of a plant-beneficial bacterial consortium. ISME J. 2018;12:1496–507. 10.1038/s41396-018-0093-1. 29520025 PMC5956071

[bib10] Berg G, Kusstatscher P, Abdelfattah A et al. Microbiome modulation—toward a better understanding of plant microbiome response to microbial inoculants. Front Microbiol. 2021;12:650610. 10.3389/fmicb.2021.650610. 33897663 PMC8060476

[bib11] Berihu M, Somera TS, Malik A et al. A framework for the targeted recruitment of crop-beneficial soil taxa based on network analysis of metagenomics data. Microbiome. 2023;11:8. 10.1186/s40168-022-01438-1. 36635724 PMC9835355

[bib13] Borriss R, Dietel K, Beifort P. Selektion und Einsatz kälte-toleranter Bacillusstämme als biologische Phytostimulatoren. Patent DE 10 2016 004 625 A1 2016.10.20, 2016.

[bib14] Bradáčová K, Florea A, Bar-Tal A et al. Microbial consortia versus single-strain inoculants: an advantage in PGPM-assisted tomato production?. Agronomy. 2019;9:2. 10.3390/agronomy9020105.

[bib15] Braun PG, Fuller KD, McRae K et al. Response of ‘Honeycrisp®’ apple trees to combinations of pre-plant fumigation, deep ripping, and hog manure compost incorporation in a soil with replant disease. HortScience. 2010;45:1702–7. 10.21273/HORTSCI.45.11.1702.

[bib17] Busnena BA, Beerhues L, Liu B. Biphenyl and dibenzofuran phytoalexins differentially inhibit root-associated microbiota in apple, including fungal and oomycetal replant disease pathogens. Phytopathology. 2025;115:181–91. 10.1094/PHYTO-03-24-0088-R. 39433045

[bib16] Busnena BA, Beerhues L, Liu B. Biphenyls and dibenzofurans of the rosaceous subtribe Malinae and their role as phytoalexins. Planta. 2023;258:78. 10.1007/s00425-023-04228-7. 37689618 PMC10492887

[bib18] Busnena BA, Beuerle T, Mahnkopp-Dirks F et al. Formation and exudation of biphenyl and dibenzofuran phytoalexins by roots of the apple rootstock M26 grown in apple replant disease soil. Phytochem. 2021;192:112972. 10.1016/j.phytochem.2021.112972. 34624729

[bib19] Callahan BJ, McMurdie PJ, Rosen MJ et al. DADA2: high-resolution sample inference from Illumina amplicon data. Nat Methods. 2016;13:581–3. 10.1038/nmeth.3869. 27214047 PMC4927377

[bib20] Chenu C, Cosentino D. Microbial regulation of soil structural dynamics. In: The Architecture and Biology of Soils—Life in Inner Space. Cambridge: CABI. 2011, 37–70.

[bib21] Chuberre C, Plancot B, Driouich A et al. Plant immunity is compartmentalized and specialized in roots. Front Plant Sci. 2018;9:1692. 10.3389/fpls.2018.01692. 30546372 PMC6279857

[bib22] Compant S, Cassan F, Kostić T et al. Harnessing the plant microbiome for sustainable crop production. Nat Rev Microbiol. 2025;23:9–23. 10.1038/s41579-024-01079-1. 39147829

[bib23] Dabney SM, Delgado JA, Reeves DW. Using winter cover crops to improve soil and water quality. Commun Soil Sci Plant Anal. 2001;32:1221–50. 10.1081/CSS-100104110.

[bib25] De Corato U. Soil microbiota manipulation and its role in suppressing soil-borne plant pathogens in organic farming systems under the light of microbiome-assisted strategies. Chem Biol Technol Agric. 2020;7:1. 10.1186/s40538-020-00183-7.

[bib27] de Mendiburu Delgado F , agricolae: statistical procedures for agricultural research. R package version 1.4.0. CRAN, 2020. https://myaseen208.github.io/agricolae/https://cran.r-project.org/package=agricolae (21 February 2025, date last accessed).

[bib28] De Souza RSC, Armanhi JSL, Arruda P. From microbiome to traits: designing synthetic microbial communities for improved crop resiliency. Front Plant Sci. 2020;11:1179. 10.3389/fpls.2020.01179. 32983187 PMC7484511

[bib29] De Tender C, Vandecasteele M, Ommeslag S et al. *Linnemannia elongata*: a key species in chitin-based plant growth promotion. Phytobiomes J. 2024;8:366–77. 10.1094/PBIOMES-05-23-0031-R.

[bib24] Debode J, de Tender C, Soltaninejad S et al. Chitin mixed in potting soil alters lettuce growth, the survival of zoonotic bacteria on the leaves and associated rhizosphere microbiology. Front Microbiol. 2016;7:565. 10.3389/fmicb.2016.00565. 27148242 PMC4838818

[bib26] Delavaux CS, Ramos RJ, Sturmer SL et al. Environmental identification of arbuscular mycorrhizal fungi using the LSU rDNA gene region: an expanded database and improved pipeline. Mycorrhiza. 2022;32:145–53. 10.1007/s00572-022-01068-3. 35099622 PMC8907093

[bib30] Ding G-C, Heuer H, Zühlke S et al. Soil type-dependent responses to phenanthrene as revealed by determining the diversity and abundance of polycyclic aromatic hydrocarbon ring-hydroxylating dioxygenase genes by using a novel PCR detection system. Appl Environ Microbiol. 2010;76:4765–71. 10.1128/AEM.00047-10. 20495045 PMC2901735

[bib31] Duan Y, Chen R, Zhang R et al. Isolation and identification of *Bacillus vallismortis* HSB-2 and its biocontrol potential against apple replant disease. Biol Contr. 2022;170:104921. 10.1016/j.biocontrol.2022.104921.

[bib32] Eltlbany N, Baklawa M, Ding G-C et al. Enhanced tomato plant growth in soil under reduced P supply through microbial inoculants and microbiome shifts. FEMS Microbiol Ecol. 2019;95:9. 10.1093/femsec/fiz124. 31386159

[bib33] FAO & ITPS . Status of the World’s Soil Resources (SWSR). Main report. Rome: FAO, 2015.

[bib34] Fierer N. Embracing the unknown: disentangling the complexities of the soil microbiome. Nat Rev Microbiol. 2017;15:579–90. 10.1038/nrmicro.2017.87. 28824177

[bib35] Finkel OM, Castrillo G, Herrera Paredes S et al. Understanding and exploiting plant beneficial microbes. Curr Opin Plant Biol. 2017;38:155–63. 10.1016/j.pbi.2017.04.018. 28622659 PMC5561662

[bib36] Freund L, Mariotte P, Santonja M et al. Species identity, rather than species mixtures, drives cover crop effects on nutrient partitioning in unfertilized agricultural soil. Plant Soil. 2021;460:149–62. 10.1007/s11104-020-04782-z.

[bib37] Gentsch N, Boy J, Batalla JDK et al. Catch crop diversity increases rhizosphere carbon input and soil microbial biomass. Biol Fertil Soil. 2020;56:943–57. 10.1007/s00374-020-01475-8.

[bib38] Gentsch N, Don A, Peth S et al. Cover crops improve soil structure and change organic carbon distribution in macroaggregate fractions. Soil. 2024;10:139–50. 10.5194/soil-10-139-2024.

[bib39] Gonçalves OS, Creevey CJ, Santana MF. Designing a synthetic microbial community through genome metabolic modeling to enhance plant-microbe interaction. Environ Microbiome. 2023;18:81. 10.1186/s40793-023-00536-3. 37974247 PMC10655421

[bib40] Hafiz FB, Moradtalab N, Goertz S et al. Synergistic effects of a root-endophytic *Trichoderma* fungus and *Bacillus* on early root colonization and defense activation against *Verticillium longisporum* in rapeseed. MPMI. 2022;35:380–92. 10.1094/MPMI-11-21-0274-R. 35147443

[bib41] Hanschen FS, Winkelmann T. Biofumigation for fighting replant disease- a review. Agronomy. 2020;10:425. 10.3390/agronomy10030425.

[bib42] Hauschild K, Orth N, Liu B et al. Rhizosphere competent inoculants modulate the apple root-associated microbiome and plant phytoalexins. Appl Microbiol Biotechnol. 2024;108:344. 10.1007/s00253-024-13181-8. 38801472 PMC11129989

[bib43] Henfrey JL, Baab G, Schmitz M. Physiological stress responses in apple under replant conditions. Sci Hortic. 2015;194:111–7. 10.1016/j.scienta.2015.07.034.

[bib44] Hett J, Döring TF, Bevivino A et al. Impact of microbial consortia on organic maize in a temperate climate varies with environment but not with fertilization. Eur J Agron. 2023;144:126743. 10.1016/j.eja.2023.126743.

[bib45] Heuermann D, Koch L, Fricke T et al. Catch crop mixtures have higher potential for nutrient carry-over than pure stands under changing environments. Eur J Agron. 2022;136:126519. 10.1016/j.eja.2022.126504.

[bib46] Hinsinger P, Gobran GR, Gregory PJ et al. Rhizosphere geometry and heterogeneity arising from root-mediated physical and chemical processes. New Phytol. 2005;168:293–303. 10.1111/j.1469-8137.2005.01512.x. 16219069

[bib47] Hooks CR, Wang K-H, Ploeg A et al. Using marigold (*Tagetes* spp.) as a cover crop to protect crops from plant-parasitic nematodes. Appl Soil Ecol. 2010;46:307–20. 10.1016/j.apsoil.2010.09.005.

[bib48] Ihrmark K, Bödeker ITM, Cruz-Martinez K et al. New primers to amplify the fungal ITS2 region—Evaluation by 454-sequencing of artificial and natural communities. FEMS Microbiol Ecol. 2012;82:666–77. 10.1111/j.1574-6941.2012.01437.x. 22738186

[bib49] Jacoby R, Peukert M, Succurro A et al. The role of soil microorganisms in plant mineral nutrition -current knowledge and future directions. Front Plant Sci. 2017;8:1617. 10.3389/fpls.2017.01617. 28974956 PMC5610682

[bib50] Jiang F, Zhang L, Zhou J et al. Arbuscular mycorrhizal fungi enhance mineralisation of organic phosphorus by carrying bacteria along their extraradical hyphae. New Phytol. 2021;230:304–15. 10.1111/nph.17081. 33205416

[bib51] Jiang W, Chen R, Zhao L et al. Chemical fumigants control apple replant disease: microbial community structure-mediated inhibition of *Fusarium* and degradation of phenolic acids. J Hazard Mater. 2022;440:129786. 10.1016/j.jhazmat.2022.129786. 36007363

[bib52] Kaloterakis N, Giongo A, Braun-Kiewnick A et al. Rotational diversity shapes the bacterial and archaeal communities and confers positive plant-soil feedback in winter wheat rotations. Soil Biol Biochem. 2025;203:109729. 10.1016/j.soilbio.2025.109729.

[bib53] Kanfra X, Obawolu T, Wrede A et al. Alleviation of nematode-mediated apple replant disease by pre-cultivation of *Tagetes*. Horticulturae. 2021;7:433. 10.3390/horticulturae7110433.

[bib54] Kaur S, Samota MK, Choudhary M et al. How do plants defend themselves against pathogens—biochemical mechanisms and genetic interventions. Physiol Mol Biol Plants. 2022;28:485–504. 10.1007/s12298-022-01146-y. 35400890 PMC8943088

[bib55] Koch L, Pausch J, Don A. Catch crop residue incorporation affects microbial nutrient dynamics in temperate arable soils. Biol Fertil Soil. 2020;56:777–90.

[bib56] Kolaříková Z, Slavíková R, Krüger C et al. PacBio sequencing of glomeromycota rDNA: a novel amplicon covering all widely used ribosomal barcoding regions and its applicability in taxonomy and ecology of arbuscular mycorrhizal fungi. New Phytol. 2021;231:490–9. 10.1111/nph.17372. 33780549

[bib57] Kolde R. pheatmap: pretty heatmaps. R package version 1.0.12. CRAN, 2019. https://CRAN.R-project.org/package=pheatmap (21 February 2025, date last accessed).

[bib58] Kublik S, Gschwendtner S, Magritsch T et al. Microplastics in soil induce a new microbial habitat, with consequences for bulk soil microbiomes. Front Environ Sci. 2022;10:989267. 10.3389/fenvs.2022.989267.

[bib59] Kuzmanović N, Eltlbany N, Ding G et al. Analysis of the genome sequence of plant beneficial strain *Pseudomonas* sp. RU47. J Biotechnol. 2018;281:183–92. 10.1016/j.jbiotec.2018.07.023. 30031092

[bib60] Li C, Zhao Q, Gao T et al. The mitigation effects of exogenous melatonin on replant disease in apple. J Pineal Res. 2018;65:e12523. 10.1111/jpi.12523. 30230015

[bib61] Li X, Chou M-Y, Bonito GM et al. Anti-fungal bioactive terpenoids in the bioenergy crop switchgrass (*Panicum virgatum*) may contribute to ecotype-specific microbiome composition. Commun Biol. 2023;6:917. 10.1038/s42003-023-05290-3. 37679469 PMC10485007

[bib62] Li X, Chu Y, Jia Y et al. Changes to bacterial communities and soil metabolites in an apple orchard as a legacy effect of different intercropping plants and soil management practices. Front Microbiol. 2022;13:956840. 10.3389/fmicb.2022.956840. 36003931 PMC9393497

[bib63] Love MI, Huber W, Anders S. Moderated estimation of fold change and dispersion for RNA-seq data with DESeq2. Genome Biol. 2014;15:550. 10.1186/s13059-014-0550-8. 25516281 PMC4302049

[bib64] Mahnkopp F, Simon M, Lehndorff E et al. Induction and diagnosis of apple replant disease (ARD): a matter of heterogeneous soil properties?. Sci Hortic. 2018;241:167–77. 10.1016/j.scienta.2018.06.076.

[bib65] Manning P, van der Plas F, Soliveres S et al. Redefining ecosystem multifunctionality. Nat Ecol Evol. 2018;2:427–36. 10.1038/s41559-017-0461-7. 29453352

[bib66] Martinez Arbizu P. pairwiseAdonis: Pairwise multilevel comparison using adonis. R package version 0.4. GitHub, 2020. https://github.com/pmartinezarbizu/pairwiseAdonis/tree/master (01 June 2025, date last accessed).

[bib67] Mawarda PC, Le Roux X, van Elsas DJ et al. Deliberate introduction of invisible invaders: a critical appraisal of the impact of microbial inoculants on soil microbial communities. Soil Biol Biochem. 2020;148:107874. 10.1016/j.soilbio.2020.107874.

[bib68] Mazzola M, Hewavitharana SS, Strauss SL. *Brassica* seed meal soil amendments transform the rhizosphere microbiome and improve apple production through resistance to pathogen reinfestation. Phytopathol. 2015;105:460–9. 10.1094/PHYTO-09-14-0247-R. 25412009

[bib69] McMurdie PJ, Holmes S. phyloseq: an R package for reproducible interactive analysis and graphics of microbiome census data. PLoS One. 2013;8:e61217. 10.1371/journal.pone.0061217. 23630581 PMC3632530

[bib70] Morgan BST, Egerton-Warburton LM. Barcoded NS31/AML2 primers for sequencing of arbuscular mycorrhizal communities in environmental samples. Appl Plant Sci. 2017;5:1700017. 10.3732/apps.1700017 PMC5584815 28924511

[bib71] Murashige T, Skoog F. A revised medium for rapid growth and bio assays with tobacco tissue cultures. Physiol Plant. 1962;15:473–97. 10.1111/j.1399-3054.1962.tb08052.x.

[bib72] Nilsson RH, Anslan S, Bahram M et al. Mycobiome diversity: high-throughput sequencing and identification of fungi. Nat Rev Microbiol. 2019;17:95–109. 10.1038/s41579-018-0116-y. 30442909

[bib73] Nwokolo NL, Enebe MC, Chigor CB et al. The contributions of biotic lines of defence to improving plant disease suppression in soils: a review. Rhizosphere. 2021;19:100372. 10.1016/j.rhisph.2021.100372.

[bib74] Oksanen J, Blanchet FG, Friendly M et al. vegan: community ecology package. R package version 2.5-7. CRAN, 2020. https://CRAN.R-project.org/package=vegan (23 February 2025, date last accessed).

[bib75] Philippot L, Raaijmakers JM, Lemanceau P et al. Going back to the roots: the microbial ecology of the rhizosphere. Nat Rev Microbiol. 2013;11:789–99. 10.1038/nrmicro3109. 24056930

[bib76] Piasecka A, Jedrzejczak-Rey N, Bednarek P. Secondary metabolites in plant innate immunity: conserved function of divergent chemicals. New Phytol. 2015;206:948–64. 10.1111/nph.13325. 25659829

[bib77] Popp C, Wamhoff D, Winkelmann T et al. Molecular identification of *Nectriaceae* in infections of apple replant disease affected roots collected by Harris Uni-Core punching or laser microdissection. J Plant Dis Prot. 2020;127:571–82. 10.1007/s41348-020-00333-x.

[bib78] Proctor C, He Y. Quantifying root extracts and exudates of sedge and shrub in relation to root morphology. Soil Biol Biochem. 2017;114:168–80. 10.1016/j.soilbio.2017.07.006.

[bib79] Quast C, Pruesse E, Yilmaz P et al. The SILVA ribosomal RNA gene database project: improved data processing and web-based tools. Nucleic Acids Res. 2013;41:D590–6. 10.1093/nar/gks1219. 23193283 PMC3531112

[bib80] R Core Team . R: a language and environment for statistical computing. Vienna: R Foundation for Statistical Computing, 2023.

[bib81] R Studio Team . RStudio: integrated Development for R. Boston, MA, 2023.

[bib82] Ranaldo M, Carlesi S, Costanzo S et al. Functional diversity of cover crop mixtures enhances biomass yield and weed suppression in a Mediterranean agroecosystem. Weed Res. 2020;60:96–108. 10.1111/wre.12388.

[bib83] Reinhold-Hurek B, Bünger W, Burbano CS et al. Roots shaping their microbiome: global hotspots for microbial activity. Ann Rev Phytopathol. 2015;53:403–24. 10.1146/annurev-phyto-082712-102342. 26243728

[bib84] Rohr A-D, Schimmel J, Liu B et al. Identification and validation of early genetic biomarkers for apple replant disease. PLoS One. 2020;15:e0238876. 10.1371/journal.pone.0238876. 32970702 PMC7514092

[bib85] Schloss PD. Rarefaction is currently the best approach to control for uneven sequencing effort in amplicon sequence analyses. mSphere. 2024;9:e0035423. 10.1128/msphere.00354-23. 38251877 PMC10900887

[bib86] Schreiter S, Babin D, Smalla K et al. Rhizosphere competence and biocontrol effect of *Pseudomonas* sp. RU47 independent from plant species and soil type at the field scale. Front Microbiol. 2018;9:97. 10.3389/fmicb.2018.00097. 29449832 PMC5799239

[bib87] Seitz VA, McGivern BB, Borton MA et al. Cover crop root exudates impact soil microbiome functional trajectories in agricultural soils. Microbiome. 2024;12:183. 10.1186/s40168-024-01886-x. 39342284 PMC11439266

[bib88] Sella SRBR, Vandenberghe LPS, Soccol CR. *Bacillus atrophaeus*: main characteristics and biotechnological applications—a review. Crit Rev Biotechnol. 2015;35:533–45. 10.3109/07388551.2014.922915. 24963702

[bib89] Sharma NC, Verma P, Singh N et al. Causes and control measures of apple replant problem. Int J Bio Resour Stress Manag. 2020a;11:246–57. 10.23910/1.2020.2090.

[bib90] Sharma S, Compant S, Ballhausen M-B et al. The interaction between *Rhizoglomus irregulare* and hyphae attached phosphate solubilizing bacteria increases plant biomass of *Solanum lycopersicum*. Microbiol Res. 2020b;240:126556. 10.1016/j.micres.2020.126556. 32683279

[bib91] Siefen N, Staudt J, Busnena BA et al. Differential accumulation of phenolics and phytoalexins in seven *Malus* genotypes cultivated in apple replant disease-affected soil. Sci Hortic. 2024;328:112902. 10.1016/j.scienta.2024.112902.

[bib92] Smith SE, Smith FA. Roles of arbuscular mycorrhizas in plant nutrition and growth: new paradigms from cellular to ecosystem scales. Ann Rev Plant Biol. 2011;62:227–50. 10.1146/annurev-arplant-042110-103846. 21391813

[bib93] Somera TS, Mazzola M. Toward a holistic view of orchard ecosystem dynamics: a comprehensive review of the multiple factors governing development or suppression of apple replant disease. Front Microbiol. 2022;13:949404. 10.3389/fmicb.2022.949404. 35958152 PMC9358454

[bib94] Strickland MS, Thomason WE, Avera B et al. Short-term effects of cover crops on soil microbial characteristics and biogeochemical processes across actively managed farms. Agrosyst Geosci Environ. 2019;2:1–9. 10.2134/age2018.12.0064.

[bib95] Sundberg C, Al-Soud WA, Larsson M et al. 454 pyrosequencing analyses of bacterial and archaeal richness in 21 full-scale biogas digesters. FEMS Microbiol Ecol. 2013;85:612–26. 10.1111/1574-6941.12148. 23678985

[bib96] Tedersoo L, Bahram M, Zinger L et al. Best practices in metabarcoding of fungi: from experimental design to results. Mol Ecol. 2022;31:2769–95. 10.1111/mec.16460. 35395127

[bib97] Tewoldemedhin YT, Mazzola M, Labuschagne I et al. A multi-phasic approach reveals that apple replant disease is caused by multiple biological agents, with some agents acting synergistically. Soil Biol Biochem. 2011;43:1917–27. 10.1016/j.soilbio.2011.05.014.

[bib98] Timmusk S, Behers L, Muthoni J et al. Perspectives and challenges of microbial application for crop improvement. Front Plant Sci. 2017;8:49. 10.3389/fpls.2017.00049. 28232839 PMC5299024

[bib99] Uroz S, Courty PE, Oger P. Plant symbionts are engineers of the plant-associated microbiome. Trends Plant Sci. 2019;24:905–16. 10.1016/j.tplants.2019.06.008. 31288964

[bib100] Utkhede RS, Sholberg PL, Smirle MJ. Effects of chemical and biological treatments on growth and yield of apple trees planted in *Phytophthora cactorum* infected soil. Canad J Plant Pathol. 2001;23:163–7. 10.1080/07060660109506925.

[bib101] van der Heijden MGA, Wagg C. Soil microbial diversity and agro-ecosystem functioning. Plant Soil. 2013;363:1–5. 10.1007/s11104-012-1545-4.

[bib102] van der Putten WH, Bardgett RD, Bever JD et al. Plant–soil feedbacks: the past, the present and future challenges. J Ecol. 2013;101:265–76. 10.1111/1365-2745.12054.

[bib103] Vukicevich E, Lowery T, Bowen P et al. Cover crops to increase soil microbial diversity and mitigate decline in perennial agriculture. A review. Agron Sustain Dev. 2016;36:48. 10.1007/s13593-016-0385-7.

[bib104] Wagg C, Bender SF, Widmer F et al. Soil biodiversity and soil community composition determine ecosystem multifunctionality. Proc Natl Acad Sci. 2014;111:5266–70. 10.1073/pnas.1320054111. 24639507 PMC3986181

[bib105] Wang H, Zhang R, Mao Y et al. Effects of *Trichoderma asperellum* 6S-2 on apple tree growth and replanted soil microbial environment. J Fungi. 2022;8:63. 10.3390/jof8010063. PMC8778220 35050003

[bib106] Wang T, Ruan Y, Xu Q et al. Effect of plant-derived microbial soil legacy in a grafting system-a turn for the better. Microbiome. 2024a;12:234. 10.1186/s40168-024-01938-2. 39543707 PMC11566652

[bib107] Wang Z, Huang Y-H, He M et al. Root-associated bacterial communities of vegetable *Brassica parachinensis* enrich pollutant-degrading taxa and functions for enhancing phthalate dissipation. Appl Soil Ecol. 2024b;202:105617. 10.1016/j.apsoil.2024.105617.

[bib108] Warmink JA, Nazir R, Corten B et al. Hitchhikers on the fungal highway: the helper effect for bacterial migration via fungal hyphae. Soil Biol Biochem. 2011;43:760–5. 10.1016/j.soilbio.2010.12.009.

[bib119_755_011625] Weiß S, Bartsch M, Winkelmann T. Transcriptomic analysis of molecular responses in Malus domestica ‘M26’ roots affected by apple replant disease. Plant Mol Biol. 2017; 94:303–318. 10.1007/s11103-017-0608-6 28424966

[bib109] Wendling M, Büchi L, Amosse C et al. Nutrient accumulation by cover crops with different root systems. Asp Appl Biol. 2015;129:91–6.

[bib110] White TJ, Bruns T, Lee S et al. Amplification and direct sequencing of fungal ribosomal RNA genes for phylogenetics. In: PCR Protocols: a Guide to Methods and Applications. Cambridge: Academic Press, 1990, 315–22.

[bib111] Wickham H. ggplot2: Elegrant Graphics for Data Analysis. New York, NY: Springer Verlag, 2016.

[bib112] Wilhelm RC, Amsili JP, Kurtz KSM et al. Ecological insights into soil health according to the genomic traits and environment-wide associations of bacteria in agricultural soils. ISME Commun. 2023;3:1. 10.1038/s43705-022-00209-1. 37081121 PMC9829723

[bib113] Winkelmann T, Smalla K, Amelung W et al. Apple replant disease: causes and mitigation strategies. Curr Iss Mol Biol. 2019;30:89–106. 10.21775/cimb.030.089. 30070653

[bib114] Wittenmayer L, Szabó K. The role of root exudates in specific apple (*Malus x domestica* Borkh.) replant disease (SARD). J Plant Nutr Soil Sci. 2000;163:399–404. 10.1002/1522-2624(200008)163:4<399::AID-JPLN399>3.0.CO;2-8.

[bib115] Yim B, Hanschen FS, Wrede A et al. Effects of biofumigation using *Brassica juncea* and *Raphanus sativus* in comparison to disinfection using Basamid on apple plant growth and soil microbial communities at three field sites with replant disease. Plant Soil. 2016;406:389–408. 10.1007/s11104-016-2876-3.

[bib116] Yim B, Heider MA, Bloem E et al. Exploring the potential of seed inoculation with microbial consortia to mitigate drought stress in maize plants under greenhouse conditions. Plant Soil. 2025. 10.1007/s11104-024-07110-x.

[bib117] Yim B, Nitt H, Wrede A et al. Effects of soil pre-treatment with Basamid® granules, *Brassica juncea, Raphanus sativus*, and *Tagetes patula* on bacterial and fungal communities at two apple replant disease sites. Front Microbiol. 2017;8:1604. 10.3389/fmicb.2017.01604. 28919882 PMC5586068

[bib118] Zhang K, Bonito G, Hsu C-H et al. *Mortierella elongata* increases plant biomass among non-leguminous crop species. Agronomy. 2020;10:754. 10.3390/agronomy10050754.

