# Supplementary material for: Catch crop amendments and microbial inoculants differently modulate apple rhizosphere microbiomes and plant responses
Source: FEMS Microbiol Ecol. 2025 May 23;101(6):fiaf055. doi: 10.1093/femsec/fiaf055 (PMC12168782; doi:10.1093/femsec/fiaf055)
Supplement: fiaf055_Supplemental_Files [file fiaf055_supplemental_files.zip › File_S1_Hauschild_et_al.docx]

SUPPLEMENTAL FILE S1

Catch crop amendments and microbial inoculants for sustainable soil health management – a greenhouse case study with apple plants

**Kristin Hauschild, Adriana Giongo, Benye Liu, Doreen Babin, Elke Bloem, Ludger Beerhues, Traud Winkelmann and Kornelia Smalla**

**Measurement of soil dehydrogenase activity, CO_2_-respiration, and C/N ratio in soils at experiment set-up.**

Soil dehydrogenase was measured using standardized procedures following DIN EN ISO 23753-2. Briefly, the soil was treated with iodotetrazoliumchlorid, incubated for 4-6 h, and subsequently the iodonitrotetrazoliumformazan was extracted with acetone. The adsorption was determined at 485 nm using a UV-VIS spectrophotometer (Lambda-35, Perkin Elmer, Rodgau, Germany). The soil CO_2_-respiration was analyzed according to DIN EN ISO 16072. Briefly, the CO_2_-release was measured by precipitation in NaOH and subsequent titration. Phenolphthalein served as a titration indicator. Total carbon (C) and nitrogen (N) were determined in air dried soil samples via dry combustion using a CN analyser (Vario MAX Cube, ELEMENTAR, Langenselbold, Germany).

**Catch crop amendments stimulated initial soil microbial activity**

At the start of the experiment, a significant increase in soil microbial activity was observed in soils amended with Tag and CCM compared to the Ctl (Tab. S1). Dehydrogenase activity was significantly higher in Tag (0.98±0.07 U) and CCM (1.49±0.2 U) compared to the Ctl (0.19±0.08 U). As expected, the soils directly sampled after the addition of microbial inoculants did not differ from Ctl soil in dehydrogenase activity. Soil carbon respiration was significantly higher in CCM (3.29 mg CO_2_ g^-1^ h^-1^) compared to the Ctl (0.45 mg CO_2_ g^-1^ h^-1^) soil. The Tag and microbial amendments led to a slight but not significant increase in carbon respiration compared to the Ctl (Tab. S1). The C/N ratio was significantly lower in soil amended with CCM (ratio 15.33±0.15) compared to the Ctl (ratio 17.18±0.3), while no differences in Tag were observed. As expected, no effect of the beneficial microbes was observed directly after their inoculation.
