# Supplementary material for: Catch crop amendments and microbial inoculants differently modulate apple rhizosphere microbiomes and plant responses
Source: FEMS Microbiol Ecol. 2025 May 23;101(6):fiaf055. doi: 10.1093/femsec/fiaf055 (PMC12168782; doi:10.1093/femsec/fiaf055)
Supplement: fiaf055_Supplemental_Files [file fiaf055_supplemental_files.zip › Supplemental_materials_revised.docx]

SUPPLEMENTAL MATERIALS

Catch crop amendments and microbial inoculants differently modulate apple rhizosphere microbiomes and plant responses

**Kristin Hauschild, Adriana Giongo, Benye Liu, Doreen Babin, Elke Bloem, Ludger Beerhues, Traud Winkelmann and Kornelia Smalla**

**Table S1:** Average values and standard errors (n=3) of dehydrogenase activity, carbon respiration (CO_2_ Resp.) and C/N ratio at set-up of the experiment in untreated ARD soil (Ctl), ARD soil amended with *Tagetes patula* (Tag), a catch crop mixture (CCM) or inoculated with beneficial bacteria (BB), arbuscular mycorrhiza (AM) or a synthetic community comprised of BB and AM (SynC). Different letters indicate significant differences between treatments (*p*<0.05) according to one-way ANOVA and post-hoc Tukey’s HSD test. Letters in bold indicate significant differences to Ctl.

|  | **Ctl** | **Tag** | **CCM** | **BB** | **AM** | **SynC** |
| --- | --- | --- | --- | --- | --- | --- |
| **Dehydrogenase activity** [U] | 0.19±0.08  c | 0.98±0.071 **b** | 1.49±0.19 **a** | 0.31±0.07 c | 0.22±0.06 c | 0.41±0.03 c |
| **CO_2_ Resp.** [mg CO_2_/g/h] | 0.45±0.07 b | 0.82±0.04 b | 3.29±0.27 **a** | 0.87±0.27 b | 0.71±0.11 b | 0.94±0.19 b |
| **C/N ratio** | 17.18±0.30 a | 16.70±0.22 a | 15.33±0.15 **b** | 17.28±0.13 a | 16.96±0.29 a | 16.94±0.12 a |

**Table S2:** CFU counts of ABi05 cells in rhizosphere (RS) from upper basal roots (BU), lower basal roots (BL), or root tips (RT) and root-affected soil (RA) of apple M.26 grown in ARD affected soils for seven weeks after treatment with beneficial bacteria (BB) or a synthetic community (SynC).

|  | **BB** | | | **SynC** | | | |
| --- | --- | --- | --- | --- | --- | --- | --- |
| **[CFU g^-1^ soil]** | **ABi05 – total cells** | **ABi05 - spores** | **ABi05 - vital cells** | | **ABi05 – total cells** | **ABi05 - spores** | **ABi05 - vital cells** |
| **RS-BU** | 9.66E+06 | 2.37E+05 | 9.42E+06 | | 8.31E+06 | 2.28E+05 | 8.08E+06 |
| **RS-BL** | 9.49E+06 | 3.84E+05 | 9.11E+06 | | 6.91E+06 | 1.75E+05 | 6.73E+06 |
| **RS-RT** | 4.07E+06 | 1.20E+05 | 3.95E+06 | | 2.94E+06 | 5.62E+04 | 2.89E+06 |
| **RA** | 8.02E+06 | 3.14E+04 | 7.99E+06 | | 8.25E+06 | 9.29E+04 | 8.16E+06 |

**Table S3:** Dissimilarity of bacterial (n=6) and fungal (n=5) communities in rhizosphere or root-affected soil of apple M.26 after growth in ARD-affected soil for 42 days under different treatments: Ctl: untreated ARD soil; Tag: amendment of *Tagetes patula*; CCM: amendment of a catch crop mixture; BB: inoculation of beneficial bacteria; AM: inoculation of arbuscular mycorrhiza; SynC: inoculation of a synthetic community comprised of BB and AM. The significance of pairwise dissimilarity between ARD and treatments Tag, CCM, BB, AM, or SynC was tested by pairwise PERMANOVA. Differences are considered significant at *p-adj*<0.05 (Benjamini-Hochberg correction; values highlighted in bold).

| **Pairwise PERMANOVA** | | **Bacteria/Archaea** | | **Fungi** | |
| --- | --- | --- | --- | --- | --- |
|  |  | **R^2^** | ***p-adj*** | **R^2^** | ***p-adj*** |
| **Rhizosphere** | **Ctl vs. Tag** | 0.228 | **0.045** | 0.475 | **0.045** |
|  | **Ctl vs. CCM** | 0.297 | **0.045** | 0.469 | **0.045** |
|  | **Ctl vs. BB** | 0.224 | **0.030** | 0.198 | 0.075 |
|  | **Ctl vs. AM** | 0.220 | **0.045** | 0.253 | **0.015** |
|  | **Ctl vs. SynC** | 0.397 | **0.015** | 0.381 | 0.090 |
| **Root-affected soil** | **Ctl vs. Tag** | 0.167 | **0.030** | 0.411 | **0.045** |
|  | **Ctl vs. CCM** | 0.212 | **0.030** | 0.468 | 0.090 |
|  | **Ctl vs. BB** | 0.136 | 0.060 | 0.151 | 0.225 |
|  | **Ctl vs. AM** | 0.184 | 0.060 | 0.151 | 0.750 |
|  | **Ctl vs. SynC** | 0.233 | **0.030** | 0.138 | 1.000 |

**Table S4:** Average relative abundance (RA; n=6) of bacterial/archaeal phyla in rhizosphere and root-affected soil of M.26 grown for 42 days in ARD soil under different treatments (n=6): Ctl: untreated ARD soil; Tag: amendment of *Tagetes patula*; CCM: amendment of a catch crop mixture; BB: inoculation of beneficial bacteria; AM: inoculation of arbuscular mycorrhiza; SynC: inoculation of a synthetic community comprised of BB and AM. Differential abundance testing was done for each treatment compared to Ctl by negative binomial distribution using DeSeq2. Phyla were considered differentially abundant to Ctl for *p-adj* <0.05 (Benjamini-Hochberg correction). Post-analysis, taxa were filtered for RA>0.5% and Log2FC <-1/>+1. LFC: Log2-fold change values- Negative LFC values indicate lower abundance, and positive LFC values higher differential abundance compared to Ctl.

|  | **Phylum** | **Ctl** | **Tag** | | | **CCM** | | | **BB** | | | **AM** | | | **SynC** | | |
| --- | --- | --- | --- | --- | --- | --- | --- | --- | --- | --- | --- | --- | --- | --- | --- | --- | --- |
|  |  | **RA** | **RA** | ***p-adj*** | **LFC** | **RA** | ***p-adj*** | **LFC** | **RA** | ***p-adj*** | **LFC** | **RA** | ***p-adj*** | **LFC** | **RA** | ***p-adj*** | **LFC** |
| **Rhizosphere** | Proteobacteria | 50.12 | 48.71 |  |  | 45.45 |  |  | 50.64 | 0.007 | 0.56 | 54.31 | <0.001 | 0.61 | 42.77 | 0.038 | -0.35 |
|  | Actinobacteriota | 18.61 | 21.74 | 0.009 | 0.37 | 26.06 | <0.001 | 0.72 | 22.23 | 0.001 | 0.83 | 21.15 | <0.001 | 0.70 | 24.75 |  |  |
|  | Acidobacteriota | 6.27 | 5.23 |  |  | 5.04 |  |  | 3.98 |  |  | 4.08 |  |  | 7.41 |  |  |
|  | Bacteroidota | 7.57 | 6.31 |  |  | 6.91 |  |  | 7.70 | 0.006 | 0.55 | 7.17 |  |  | 3.56 | <0.001 | -1.24 |
|  | Chloroflexi | 2.55 | 2.34 |  |  | 2.05 |  |  | 1.56 |  |  | 2.52 | 0.002 | 0.49 | 3.60 | <0.001 | 0.37 |
|  | Firmicutes | 5.15 | 6.97 | 0.014 | 0.58 | 6.78 | <0.001 | 0.64 | 5.51 | <0.001 | 0.60 | 3.76 |  |  | 8.84 | <0.001 | 0.64 |
|  | Verrucomicrobiota | 2.33 | 2.43 |  |  | 1.95 |  |  | 3.86 | 0.001 | 1.24 | 2.64 |  |  | 2.13 |  |  |
|  | Gemmatimonadota | 2.02 | 2.28 | 0.042 | 0.33 | 2.11 |  |  | 1.33 |  |  | 1.43 |  |  | 2.83 | 0.001 | 0.35 |
|  | Myxococcota | 0.96 | 1.07 | 0.008 | 0.30 | 0.86 |  |  | 0.68 |  |  | 0.62 |  |  | 1.35 | <0.001 | 0.36 |
|  | Others | 4.41 | 2.91 |  |  | 2.80 |  |  | 2.52 |  |  | 2.32 |  |  | 2.76 |  |  |
|  | |  |  |  |  |  |  |  |  |  |  |  |  |  |  |  |  |
| **Root-affected soil** | Proteobacteria | 28.04 | 26.20 |  |  | 29.88 | 0.029 | 0.27 | 25.71 |  |  | 22.07 | 0.039 | -0.31 | 21.50 | 0.017 | -0.35 |
|  | Actinobacteriota | 24.96 | 25.48 |  |  | 25.53 | 0.002 | 0.21 | 24.64 |  |  | 23.50 |  |  | 22.28 |  |  |
|  | Acidobacteriota | 11.64 | 12.49 |  |  | 9.78 |  |  | 10.78 |  |  | 11.88 |  |  | 11.62 |  |  |
|  | Bacteroidota | 2.23 | 1.87 |  |  | 4.29 | <0.001 | 1.12 | 2.20 |  |  | 2.30 |  |  | 2.29 |  |  |
|  | Chloroflexi | 5.63 | 6.04 |  |  | 4.20 | <0.001 | -0.25 | 5.23 |  |  | 5.21 |  |  | 4.59 | 0.020 | -0.27 |
|  | Firmicutes | 14.81 | 15.08 |  |  | 15.13 | 0.035 | 0.20 | 19.12 | <0.001 | 0.49 | 20.05 | <0.001 | 0.47 | 23.60 | <0.001 | 0.71 |
|  | Verrucomicrobiota | 1.95 | 2.12 |  |  | 2.00 |  |  | 1.88 |  |  | 1.91 |  |  | 1.61 |  |  |
|  | Gemmatimonadota | 4.16 | 4.14 |  |  | 4.16 |  |  | 4.58 | 0.002 | 0.26 | 4.84 | 0.001 | 0.25 | 4.36 |  |  |
|  | Crenarchaeota | 1.61 | 0.99 | 0.001 | -0.80 | 0.76 | <0.001 | -0.90 | 1.37 |  |  | 3.48 | <0.001 | 1.12 | 2.71 | <0.001 | 0.76 |
|  | Myxococcota | 1.67 | 1.91 |  |  | 1.39 |  |  | 1.46 |  |  | 1.41 | 0.022 | -0.21 | 1.44 | 0.044 | -0.18 |
|  | Others | 3.30 | 3.68 |  |  | 2.88 |  |  | 3.03 |  |  | 3.36 |  |  | 4.00 |  |  |

**Table S5:** Average relative abundance (RA in [%]; n=6) of differentially abundant bacterial/archaeal taxa in the rhizosphere of M.26 grown for 42 days in ARD soil under different treatments (n=6): Ctl: untreated ARD soil; Tag: amendment of *Tagetes patula*; CCM: amendment of a catch crop mixture; BB: inoculation of beneficial bacteria; AM: inoculation of arbuscular mycorrhiza; SynC: inoculation of a synthetic community comprised of BB and AM. Differential abundance testing was done for each treatment compared to Ctl by negative binomial distribution using DeSeq2. Phyla were considered differentially abundant to Ctl for *p-adj* <0.05 (Benjamini-Hochberg correction). Post-analysis, taxa were filtered for RA>0.5% and Log2FC <-1/>+1. LFC: Log2-fold change values. Negative LFC values indicate lower abundance and positive LFC values higher differential abundance compared to Ctl.

|  | **Taxon** | **Ctl** | **Tag** | | | **CCM** | | | **BB** | | | **AM** | | | **SynC** | | |
| --- | --- | --- | --- | --- | --- | --- | --- | --- | --- | --- | --- | --- | --- | --- | --- | --- | --- |
|  |  | **RA** | **RA** | ***p-adj*** | **LFC** | **RA** | ***p-adj*** | **LFC** | **RA** | ***p-adj*** | **LFC** | **RA** | ***p-adj*** | **LFC** | **RA** | ***p-adj*** | **LFC** |
| **Rhizosphere** | *Acidibacter* | 0.17 | 0.21 |  |  | 0.16 |  |  | 0.13 |  |  | 0.23 | 0.018 | 0.60 | 0.57 | <0.001 | 1.51 |
|  | *Arachidicoccus* | 1.21 | 0.78 |  |  | 1.03 |  |  | 0.94 |  |  | 1.61 |  |  | 0.32 | 0.002 | -2.15 |
|  | *Arthrobacter* | 0.46 | 0.61 |  |  | 0.88 | <0.001 | 1.02 | 0.38 |  |  | 0.67 |  |  | 0.34 | 0.021 | -0.65 |
|  | *Asticcacaulis* | 0.65 | 0.63 |  |  | 0.60 |  |  | 0.56 |  |  | 0.61 |  |  | 0.36 | <0.001 | -1.13 |
|  | *Conexibacter* | 0.22 | 0.29 |  |  | 0.27 |  |  | 0.27 | 0.038 | 0.66 | 0.37 | <0.001 | 0.94 | 0.68 | <0.001 | 1.51 |
|  | *Dyadobacter* | 1.44 | 0.65 | <0.001 | -1.19 | 0.46 | <0.001 | -1.60 | 2.13 | 0.010 | 0.91 | 1.40 |  |  | 0.78 | 0.001 | -1.07 |
|  | *Flavobacterium* | 0.74 | 0.58 |  |  | 0.20 | 0.001 | -1.82 | 0.34 |  |  | 0.39 |  |  | 0.02 | <0.001 | -5.25 |
|  | *Gaiella* | 0.49 | 0.41 |  |  | 0.38 |  |  | 0.31 |  |  | 0.22 | <0.001 | -1.11 | 0.66 |  |  |
|  | *Luteolibacter* | 0.33 | 0.36 |  |  | 0.29 |  |  | 0.57 | <0.001 | 1.01 | 0.54 |  |  | 0.41 |  |  |
|  | *Massilia* | 0.70 | 0.40 | <0.001 | -0.93 | 0.42 | 0.040 | -0.77 | 0.36 |  |  | 0.56 |  |  | 0.39 | <0.001 | -1.13 |
|  | *Mucilaginibacter* | 0.38 | 0.47 |  |  | 0.58 | <0.001 | 0.68 | 0.38 |  |  | 0.45 |  |  | 0.12 | <0.001 | -1.93 |
|  | *Nitrospira* | 0.68 | 0.38 | <0.001 | -0.85 | 0.34 | <0.001 | -0.90 | 0.29 | 0.031 | -0.97 | 0.13 | <0.001 | -2.28 | 0.37 | <0.001 | -1.01 |
|  | *Paenarthrobacter* | 0.14 | 0.15 |  |  | 0.32 | 0.010 | 1.27 | 0.31 | 0.033 | 1.39 | 0.82 | 0.011 | 2.69 | 0.15 |  |  |
|  | *Para_Burkholderia*  *_Caballeronia* | 4.01 | 2.97 |  |  | 3.23 |  |  | 2.90 |  |  | 4.70 |  |  | 1.82 | <0.001 | -1.36 |
|  | *Pedobacter* | 0.94 | 1.12 |  |  | 1.74 | 0.001 | 0.90 | 1.56 | 0.006 | 0.99 | 0.58 |  |  | 0.54 | <0.001 | -1.07 |
|  | *Pseudarthrobacter* | 2.13 | 4.00 | <0.001 | 0.83 | 8.15 | <0.001 | 1.93 | 2.44 |  |  | 1.71 |  |  | 2.07 |  |  |
|  | *Puia* | 0.86 | 0.69 |  |  | 0.45 |  |  | 0.30 | <0.001 | -1.20 | 0.87 |  |  | 0.16 | <0.001 | -2.61 |
|  | *RB41* | 0.52 | 0.19 | <0.001 | -1.58 | 0.17 | <0.001 | -1.49 | 0.14 | 0.007 | -1.47 | 0.04 | <0.001 | -3.51 | 0.13 | <0.001 | -2.14 |
|  | *Rhodanobacter* | 0.85 | 0.74 |  |  | 0.96 |  |  | 0.59 |  |  | 0.62 |  |  | 0.35 | <0.001 | -1.52 |
|  | *Sphingopyxis* | 1.13 | 0.56 | <0.001 | -1.16 | 0.50 | 0.001 | -1.19 | 1.06 |  |  | 1.55 |  |  | 0.85 | 0.033 | -0.69 |
|  | *Streptomyces* | 5.33 | 4.27 |  |  | 3.49 |  |  | 10.3 | 0.005 | 1.29 | 7.84 |  |  | 6.02 |  |  |
|  | *Terrabacter* | 0.16 | 0.28 | 0.006 | 0.70 | 0.68 | <0.001 | 2.14 | 0.25 | 0.002 | 0.94 | 0.15 |  |  | 0.32 | <0.001 | 0.86 |
|  | *Terrimicrobium* | 0.72 | 0.66 |  |  | 0.48 |  |  | 1.09 |  |  | 1.08 |  |  | 0.33 | 0.003 | -1.35 |
|  | *TM7a* | 0.74 | 0.12 | <0.001 | -2.61 | 0.13 | <0.001 | -2.46 | 0.36 |  |  | 0.55 |  |  | 0.12 | <0.001 | -2.80 |
|  | *Verrucomicrobium* | 0.05 | 0.14 |  |  | 0.08 |  |  | 0.96 | 0.001 | 4.50 | 0.08 |  |  | 0.17 |  |  |

**Table S6:** Average relative abundance (RA in [%]; n=6) of differentially abundant bacterial/archaeal taxa in root-affected soil of M.26 grown for 42 days in ARD soil under different treatments (n=6): Ctl: untreated ARD soil; Tag: amendment of *Tagetes patula*; CCM: amendment of a catch crop mixture; BB: inoculation of beneficial bacteria; AM: inoculation of arbuscular mycorrhiza; SynC: inoculation of a synthetic community comprised of BB and AM. Differential abundance testing was done for each treatment compared to Ctl by negative binomial distribution using DeSeq2. Phyla were considered differentially abundant to Ctl for *p-adj* <0.05 (Benjamini-Hochberg correction). Post-analysis, taxa were filtered for RA>0.5% and Log2FC <-1/>+1. LFC: Log2-fold change values. Negative LFC values indicate lower abundance and positive LFC values higher differential abundance compared to Ctl.

|  | **Taxon** | **Ctl** | **Tag** | | | **CCM** | | | **BB** | | | **AM** | | | **SynC** | | |
| --- | --- | --- | --- | --- | --- | --- | --- | --- | --- | --- | --- | --- | --- | --- | --- | --- | --- |
|  |  | **RA** | **RA** | ***p-adj*** | **LFC** | **RA** | ***p-adj*** | **LFC** | **RA** | ***p-adj*** | **LFC** | **RA** | ***p-adj*** | **LFC** | **RA** | ***p-adj*** | **LFC** |
| **Root-affected soil** | *Allo_Neo_Para_Rhizobium* | 1.83 | 0.99 | 0.025 | -0.99 | 1.72 |  |  | 0.78 | 0.014 | -1.20 | 0.68 |  |  | 0.16 | <0.001 | -3.33 |
|  | *Bacillus* | 3.99 | 3.62 |  |  | 4.36 |  |  | 6.12 | <0.001 | 0.64 | 5.08 | <0.001 | 0.47 | 7.28 | <0.001 | 1.04 |
|  | *Candidatus* Nitrocosmicus | 0.40 | 0.19 | 0.001 | -1.08 | 0.23 |  |  | 0.46 |  |  | 0.94 | <0.001 | 1.48 | 0.89 | <0.001 | 1.39 |
|  | *Candidatus* Nitrosotalea | 0.74 | 0.39 | 0.003 | -0.97 | 0.24 | <0.001 | -1.56 | 0.41 | 0.021 | -0.82 | 1.55 | 0.007 | 1.21 | 0.99 |  |  |
|  | *Devosia* | 0.44 | 0.39 |  |  | 0.64 | <0.001 | 0.60 | 0.43 |  |  | 0.27 | 0.031 | -0.63 | 0.15 | <0.001 | -1.41 |
|  | *Ligilactobacillus* | 0.00 | 0.00 |  |  | 0.00 |  |  | 0.00 |  |  | 0.22 | <0.001 | 24.66 | 0.55 | <0.001 | 11.01 |
|  | *Novosphingobium* | 0.92 | 0.69 |  |  | 1.02 |  |  | 0.69 |  |  | 0.43 |  |  | 0.07 | <0.001 | -3.63 |
|  | *Para_Burkholderia*  *_Caballeronia* | 0.59 | 0.54 |  |  | 0.68 |  |  | 0.53 |  |  | 0.30 | 0.041 | -0.83 | 0.21 | <0.001 | -1.34 |
|  | *Pedobacter* | 0.25 | 0.10 | 0.008 | -1.56 | 0.72 | <0.001 | 1.66 | 0.18 |  |  | 0.06 | 0.002 | -1.88 | 0.01 | <0.001 | -3.50 |
|  | *Pseudarthrobacter* | 1.89 | 2.34 |  |  | 5.08 | <0.001 | 1.49 | 2.76 | <0.001 | 0.57 | 2.17 |  |  | 1.24 | 0.024 | -0.42 |
|  | *Rhodanobacter* | 0.27 | 0.24 |  |  | 0.55 | <0.001 | 1.14 | 0.36 |  |  | 0.18 |  |  | 0.16 | 0.012 | -0.62 |
|  | *Terrabacter* | 0.38 | 0.27 | <0.001 | -0.54 | 0.75 | <0.001 | 1.02 | 0.50 | 0.030 | 0.37 | 0.43 |  |  | 0.31 |  |  |

**Table S7:** Average relative abundance (RA in [%]; n=5) of fungal phyla in the rhizosphere and root-affected soil of M.26 grown for 42 days in ARD soil under different treatments (n=6): Ctl: untreated ARD soil; Tag: amendment of *Tagetes patula*; CCM: amendment of a catch crop mixture; BB: inoculation of beneficial bacteria; AM: inoculation of arbuscular mycorrhiza; SynC: inoculation of a synthetic community comprised of BB and AM. Differential abundance testing was done for each treatment compared to Ctl by negative binomial distribution using DeSeq2. Phyla were considered differentially abundant to Ctl for *p-adj* <0.05 (Benjamini-Hochberg correction). Post-analysis, taxa were filtered for RA>0.5% and Log2FC <-1/>+1. LFC: Log2-fold change values. Negative LFC values indicate lower abundance and positive LFC values higher differential abundance compared to Ctl.

|  | **Phylum** | **Ctl** | **Tag** | | | **CCM** | | | **BB** | | | **AM** | | | **SynC** | | |
| --- | --- | --- | --- | --- | --- | --- | --- | --- | --- | --- | --- | --- | --- | --- | --- | --- | --- |
|  |  | **RA** | **RA** | ***p-adj*** | **LFC** | **RA** | ***p-adj*** | **LFC** | **RA** | ***p-adj*** | **LFC** | **RA** | ***p-adj*** | **LFC** | **RA** | ***p-adj*** | **LFC** |
| **Rhizo-sphere** | Ascomycota | 81.75 | 50.68 | <0.001 | -1.15 | 47.63 | <0.001 | -1.39 | 79.01 |  |  | 58.81 | 0.004 | -1.15 | 57.73 | <0.001 | -1.37 |
|  | Mortierellomycota | 8.47 | 37.52 | <0.001 | 1.84 | 39.64 | <0.001 | 1.81 | 8.12 |  |  | 23.37 | 0.004 | 1.07 | 25.12 | 0.003 | 0.96 |
|  | Basidiomycota | 8.20 | 9.53 |  |  | 9.63 |  |  | 11.85 |  |  | 16.24 |  |  | 14.60 |  |  |
|  | Others | 1.59 | 2.27 |  |  | 3.10 |  |  | 1.02 |  |  | 1.58 |  |  | 2.55 |  |  |
|  | | | | | | | | | | | | | | | | | |
| **Root-affected soil** | Ascomycota | 49.80 | 42.44 |  |  | 44.67 | <0.001 | 0.92 | 50.59 |  |  | 59.81 |  |  | 41.25 |  |  |
|  | Basidiomycota | 22.36 | 36.18 |  |  | 39.68 |  |  | 9.43 |  |  | 10.05 |  |  | 15.18 |  |  |
|  | Mortierellomycota | 23.75 | 18.63 | <0.001 | 0.96 | 13.00 | <0.001 | 1.87 | 33.87 | <0.001 | -1.40 | 21.71 | 0.004 | -1.38 | 37.90 |  |  |
|  | Mucoromycota | 0.65 | 1.71 | <0.001 | 1.66 | 2.00 | <0.001 | 2.72 | 1.51 | 0.002 | 1.11 | 5.21 | <0.001 | 0.80 | 1.21 |  |  |
|  | Others | 0.31 | 0.09 |  |  | 0.06 |  |  | 0.42 |  |  | 0.29 |  |  | 0.41 |  |  |

**Table S8:** Average relative abundance (RA in [%]; n=5) of differentially abundant fungal taxa in the rhizosphere of M.26 grown for 42 days in ARD soil under different treatments (n=6): Ctl: untreated ARD soil; Tag: amendment of *Tagetes patula*; CCM: amendment of a catch crop mixture; BB: inoculation of beneficial bacteria; AM: inoculation of arbuscular mycorrhiza; SynC: inoculation of a synthetic community comprised of BB and AM. Differential abundance testing was done for each treatment compared to Ctl by negative binomial distribution using DeSeq2. Phyla were considered differentially abundant to Ctl for *p-adj* <0.05 (Benjamini-Hochberg correction). Post-analysis, taxa were filtered for RA>0.5% and Log2FC <-1/>+1. LFC: Log2-fold change values. Negative LFC values indicate lower abundance and positive LFC values higher differential abundance compared to Ctl.

|  | **Taxon** | **Ctl** | **Tag** | | | **CCM** | | | **BB** | | | **AM** | | | **SynC** | | |
| --- | --- | --- | --- | --- | --- | --- | --- | --- | --- | --- | --- | --- | --- | --- | --- | --- | --- |
|  |  | **RA** | **RA** | ***p-adj*** | **LFC** | **RA** | ***p-adj*** | **LFC** | **RA** | ***p-adj*** | **LFC** | **RA** | ***p-adj*** | **LFC** | **RA** | ***p-adj*** | **LFC** |
| **Rhizosphere** | *Arachniotus* | 0.87 | 0.14 | <0.001 | -2.88 | 0.13 | <0.001 | -3.02 | 0.27 |  |  | 0.34 | <0.001 | -1.80 | 0.30 | <0.001 | -2.10 |
|  | *Ascobolus* | 1.08 | 0.09 | <0.001 | -3.87 | 0.09 | <0.001 | -3.89 | 0.10 | 0.005 | -3.58 | 0.09 | <0.001 | -3.91 | 0.10 | <0.001 | -4.00 |
|  | *Aspergillus* | 4.38 | 0.93 | <0.001 | -2.48 | 0.87 | <0.001 | -2.51 | 3.86 |  |  | 3.27 |  |  | 1.27 | <0.001 | -2.36 |
|  | *Beauveria* | 0.42 | 5.63 | 0.009 | 3.58 | 0.80 |  |  | 0.04 | 0.005 | -3.70 | 0.48 |  |  | 1.56 |  |  |
|  | *Clitopilus* | 0.74 | 0.19 | 0.006 | -2.22 | 0.14 | <0.001 | -2.40 | 0.63 |  |  | 0.25 | <0.001 | -1.96 | 0.23 | 0.006 | -2.24 |
|  | *Clonostachys* | 0.09 | 0.60 | <0.001 | 2.53 | 1.72 | <0.001 | 4.16 | 0.04 |  |  | 0.32 |  |  | 0.26 |  |  |
|  | *Coprinellus* | 0.01 | 0.02 |  |  | 0.16 | <0.001 | 4.07 | 0.63 | <0.001 | 6.06 | 0.21 | <0.001 | 4.19 | 0.02 |  |  |
|  | *Exophiala* | 0.11 | 0.16 |  |  | 0.30 |  |  | 0.59 | 0.006 | 2.21 | 0.33 |  |  | 0.20 |  |  |
|  | *Geomyces* | 4.22 | 0.55 | <0.001 | -3.18 | 0.67 | <0.001 | -2.86 | 1.44 |  |  | 1.66 | <0.001 | -1.72 | 1.19 | <0.001 | -2.37 |
|  | *Humicola* | 0.24 | 1.51 | 0.041 | 2.17 | 2.71 | <0.001 | 3.12 | 1.26 |  |  | 0.47 |  |  | 0.57 |  |  |
|  | *Hypomyces* | 0.003 | 0.02 | 0.017 | 2.02 | 0.04 | <0.001 | 3.28 | 1.40 | <0.001 | 8.13 | 0.04 | 0.014 | 3.06 | 0.01 |  |  |
|  | *Linnemannia* | 4.52 | 28.00 | 0.001 | 2.50 | 29.82 | <0.001 | 2.59 | 1.34 |  |  | 12.46 |  |  | 12.72 |  |  |
|  | *Minimedusa* | 0.03 | 0.01 |  |  | 0.20 | 0.001 | 2.70 | 1.08 | <0.001 | 4.93 | 0.17 | 0.011 | 2.29 | 0.02 |  |  |
|  | *Moesziomyces* | 0.30 | 0.21 |  |  | 0.10 | 0.042 | -2.01 | 0.31 |  |  | 2.33 | 0.020 | 2.53 | 0.77 |  |  |
|  | *Mortierella* | 3.18 | 8.28 | 0.009 | 1.18 | 8.71 | <0.001 | 1.29 | 6.45 |  |  | 10.14 | <0.001 | 1.32 | 11.66 | <0.001 | 1.36 |
|  | *Mucor* | 0.08 | 1.64 | <0.001 | 4.34 | 2.22 | <0.001 | 4.86 | 0.10 |  |  | 0.18 |  |  | 0.20 |  |  |
|  | *Naganishia* | 0.34 | 0.68 |  |  | 0.62 |  |  | 0.24 |  |  | 0.76 | 0.033 | 0.78 | 1.03 | 0.006 | 1.08 |
|  | *Oidiodendron* | 4.62 | 0.74 | <0.001 | -2.90 | 0.86 | <0.001 | -2.58 | 1.58 |  |  | 1.85 | 0.027 | -1.70 | 1.52 | 0.003 | -2.20 |
|  | *Penicillium* | 7.29 | 1.77 | 0.002 | -2.44 | 1.57 | <0.001 | -2.48 | 8.02 |  |  | 4.89 |  |  | 2.51 | <0.001 | -2.20 |
|  | *Pseudogymnoascus* | 17.48 | 6.58 | 0.003 | -1.78 | 9.50 | 0.027 | -1.16 | 4.09 | 0.047 | -2.12 | 4.99 | <0.001 | -2.25 | 4.62 | <0.001 | -2.57 |
|  | *Rhodotorula* | 0.18 | 1.87 | 0.003 | 3.14 | 0.38 | 0.003 | 3.14 | 0.58 |  |  | 1.44 | <0.001 | 2.61 | 2.59 | <0.001 | 3.37 |
|  | *Rozellomycota* | 0.95 | 0.15 | <0.001 | -2.95 | 0.08 | <0.001 | -3.75 | 0.28 |  |  | 0.70 |  |  | 1.55 |  |  |
|  | *Saitozyma* | 1.14 | 0.29 | <0.001 | -2.29 | 0.35 | <0.001 | -1.90 | 1.29 |  |  | 0.60 | <0.001 | -1.29 | 0.46 | <0.001 | -1.87 |
|  | *Talaromyces* | 0.08 | 0.55 | 0.002 | 2.33 | 0.26 | <0.001 | 1.34 | 0.08 |  |  | 0.10 |  |  | 0.25 |  |  |
|  | *Tetracladium* | 0.26 | 0.21 |  |  | 0.71 | 0.016 | 1.19 | 6.82 | <0.001 | 4.36 | 0.67 |  |  | 0.22 |  |  |
|  | *Trichoderma* | 9.14 | 1.96 | <0.001 | -2.52 | 1.80 | <0.001 | -2.56 | 7.63 |  |  | 6.63 |  |  | 4.34 | 0.003 | -1.67 |
|  | *Trichosporiella* | 0.16 | 0.13 |  |  | 0.34 |  |  | 5.22 | <0.001 | -1.80 | 0.34 |  |  | 0.15 |  |  |

**Table S9:** Average relative abundance (RA in [%]; n=5) of differentially abundant fungal taxa in root-affected soil of M.26 grown for 42 days in ARD soil under different treatments (n=6): Ctl: untreated ARD soil; Tag: amendment of *Tagetes patula*; CCM: amendment of a catch crop mixture; BB: inoculation of beneficial bacteria; AM: inoculation of arbuscular mycorrhiza; SynC: inoculation of a synthetic community comprised of BB and AM. Differential abundance testing was done for each treatment compared to Ctl by negative binomial distribution using DeSeq2. Phyla were considered differentially abundant to Ctl for *p-adj* <0.05 (Benjamini-Hochberg correction). Post-analysis, taxa were filtered for RA>0.5% and Log2FC <-1/>+1. LFC: Log2-fold change values. Negative LFC values indicate lower abundance and positive LFC values higher differential abundance compared to Ctl.

|  | **Taxon** | **Ctl** | **Tag** | | | **CCM** | | | **BB** | | | **AM** | | | **SynC** | | |
| --- | --- | --- | --- | --- | --- | --- | --- | --- | --- | --- | --- | --- | --- | --- | --- | --- | --- |
|  |  | **RA** | **RA** | ***p-adj*** | **LFC** | **RA** | ***p-adj*** | **LFC** | **RA** | ***p-adj*** | **LFC** | **RA** | ***p-adj*** | **LFC** | **RA** | ***p-adj*** | **LFC** |
| **Root-affected soil** | *Calophoma* | 0.50 | 0.09 | <0.001 | -1.73 | 0.20 | 0.005 | -0.62 | 0.81 |  |  | 0.55 |  |  | 0.96 |  |  |
|  | *Cephalotrichum* | 0.31 | 0.17 |  |  | 0.51 | <0.001 | 1.35 | 0.11 | 0.015 | -1.49 | 0.02 | <0.001 | -3.88 | 0.32 |  |  |
|  | *Chrysozyma* | 1.61 | 1.52 |  |  | 0.13 | <0.001 | -2.88 | 0.52 |  |  | 0.74 |  |  | 0.68 |  |  |
|  | *Cladosporium* | 0.61 | 0.99 | 0.001 | 1.30 | 1.27 | <0.001 | 1.70 | 2.34 |  |  | 0.85 |  |  | 0.58 |  |  |
|  | *Coprinellus* | 0.19 | 1.56 | <0.001 | 3.75 | 0.24 |  |  | 1.58 |  |  | 0.03 | 0.006 | -2.69 | 13.95 | <0.001 | 7.36 |
|  | *Exophiala* | 0.55 | 1.37 | <0.001 | 1.92 | 0.40 |  |  | 0.88 |  |  | 0.95 |  |  | 0.60 |  |  |
|  | *Fusicolla* | 0.46 | 0.44 |  |  | 0.60 | <0.001 | 1.04 | 1.48 | 0.002 | 1.96 | 0.46 |  |  | 0.51 |  |  |
|  | *Gibellulopsis* | 2.01 | 0.74 | <0.001 | -0.84 | 0.45 | <0.001 | -1.48 | 1.84 |  |  | 1.56 |  |  | 2.36 |  |  |
|  | *Holtermannia* | 0.91 | 0.07 | <0.001 | -3.07 | 0.06 | <0.001 | -3.39 | 0.68 |  |  | 0.32 |  |  | 3.02 | 0.007 | 1.86 |
|  | *Humicola* | 0.60 | 2.00 | <0.001 | 2.33 | 3.45 | <0.001 | 3.17 | 0.35 |  |  | 0.26 |  |  | 0.40 |  |  |
|  | *Hyalorbilia* | 0.20 | 1.25 | <0.001 | 3.29 | 0.22 |  |  | 0.11 |  |  | 0.15 |  |  | 0.16 |  |  |
|  | *Hymenoscyphus* | 1.80 | 1.30 |  |  | 1.71 | 0.014 | 0.59 | 3.44 | 0.042 | 1.27 | 2.41 |  |  | 1.86 |  |  |
|  | *Ilyonectria* | 1.57 | 1.31 |  |  | 1.64 | 0.041 | 0.73 | 0.98 |  |  | 0.82 |  |  | 0.71 | 0.006 | -1.11 |
|  | *Linnemannia* | 9.93 | 22.55 | <0.001 | 1.79 | 28.38 | <0.001 | 2.17 | 2.16 | <0.001 | -2.14 | 1.92 | <0.001 | -2.33 | 2.92 | <0.001 | -1.76 |
|  | *Minimedusa* | 0.05 | 1.14 | <0.001 | 5.31 | 0.26 | <0.001 | 3.15 | 0.35 | <0.001 | 3.11 | 0.09 |  |  | 0.53 | 0.007 | 3.30 |
|  | *Moesziomyces* | 2.33 | 0.18 | <0.001 | -3.02 | 0.05 | <0.001 | -4.89 | 1.81 |  |  | 2.91 |  |  | 0.75 |  |  |
|  | *Mucor* | 0.23 | 1.60 | <0.001 | 3.41 | 1.84 | <0.001 | 3.65 | 0.44 |  |  | 4.16 | 0.001 | 4.94 | 0.24 |  |  |
|  | *Pseudogymnoascus* | 1.98 | 4.49 | <0.001 | 1.80 | 8.20 | <0.001 | 2.72 | 1.00 | 0.037 | -0.89 | 0.99 |  |  | 0.75 | <0.001 | -1.36 |
|  | *Rhexocercosporidium* | 0.78 | 1.90 | <0.001 | 1.94 | 1.44 | <0.001 | 1.57 | 0.53 |  |  | 0.35 |  |  | 0.60 |  |  |
|  | *Rhodotorula* | 4.35 | 0.13 | <0.001 | -4.66 | 0.45 | 0.015 | -2.64 | 14.61 |  |  | 5.04 |  |  | 3.02 |  |  |
|  | *Rozellomycota* | 0.75 | 0.24 | 0.011 | -0.98 | 0.09 | <0.001 | -2.38 | 0.96 |  |  | 1.00 |  |  | 1.52 | 0.028 | 1.05 |
|  | *Sarcopodium* | 0.50 | 0.66 | <0.001 | 1.04 | 0.74 | <0.001 | 1.22 | 0.42 |  |  | 0.29 |  |  | 0.26 |  |  |
|  | *Sarocladium* | 2.64 | 0.74 | 0.001 | -1.20 | 0.88 | 0.045 | -0.87 | 3.10 |  |  | 2.62 |  |  | 2.27 |  |  |
|  | *Sclerostagonospora* | 0.31 | 0.08 | <0.001 | -1.29 | 0.09 | <0.001 | -1.06 | 0.29 |  |  | 0.89 |  |  | 0.22 |  |  |
|  | *Terramyces* | 0.001 | 0.00 |  |  | 0.001 |  |  | 1.03 | 0.018 | 9.14 | 0.02 |  |  | 0.01 |  |  |
|  | *Tetracladium* | 0.22 | 2.47 | <0.001 | 4.12 | 1.36 | <0.001 | 3.27 | 0.38 |  |  | 0.32 |  |  | 0.37 |  |  |
|  | *Trichosporiella* | 0.26 | 1.28 | <0.001 | 2.97 | 0.74 | <0.001 | 2.25 | 0.40 |  |  | 0.37 |  |  | 0.61 |  |  |
|  | *Umbelopsis* | 0.39 | 0.11 | <0.001 | -1.12 | 0.14 | <0.001 | -0.80 | 0.85 | <0.001 | 1.25 | 0.79 |  |  | 0.81 | 0.006 | 1.08 |
|  | *Zygotorulaspora* | 0.94 | 0.10 | <0.001 | -2.62 | 0.02 | <0.001 | -4.58 | 0.27 | <0.001 | -1.76 | 0.37 | 0.027 | -1.29 | 0.29 | 0.011 | -1.70 |


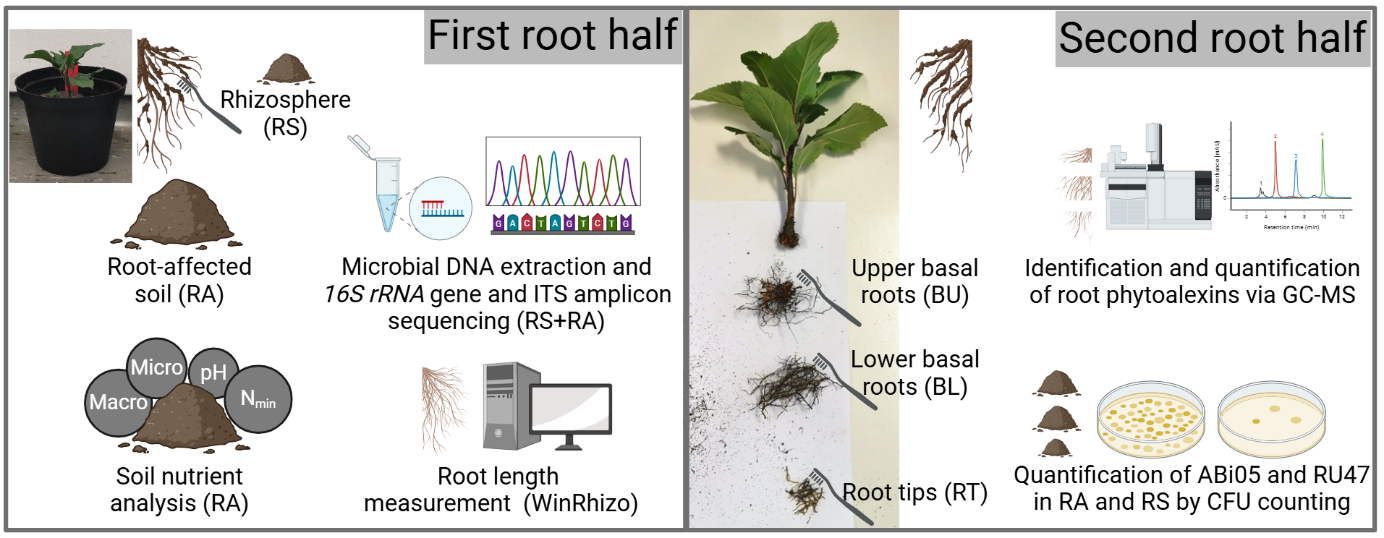


**Fig. S1:** Scheme of destructive sampling seven weeks after the setup of the experiment. M.26 plants were carefully removed from their pots, and root systems were split into halves and processed separately. Amplicon sequencing of the *16S rRNA* gene and ITS region was performed from root-affected soil and rhizosphere harvested from the first root half. CFU counts of the bacterial inoculants and root phytoalexins were determined from the second root half, separated into upper basal root (BU), lower basal root (BL), and root tips (RT).

**
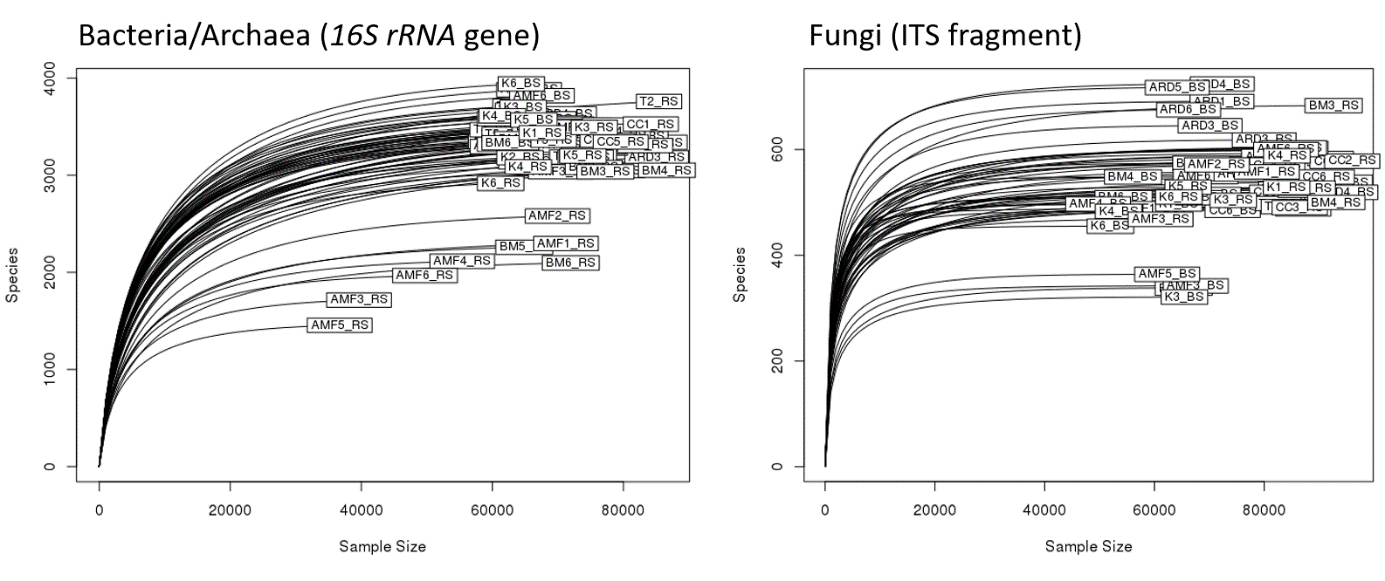
**

**Fig. S2:** Rarefaction curves of the *16S rRNA* gene and ITS fragment amplicon sequencing of DNA extracted from root-affected soil (BS, later renamed to RA) and rhizosphere (RS) of apple M.26 after growth in ARD-affected soil for 42 days under different treatments: ARD: untreated ARD soil (=Ctl); T: amendment of *Tagetes patula* (Tag); CC: amendment of a catch crop mixture (CCM); BM: inoculation of beneficial bacteria (BB); AMF: inoculation of arbuscular mycorrhiza (AM); K: inoculation of a synthetic community comprised of BM and AMF (=SynC).


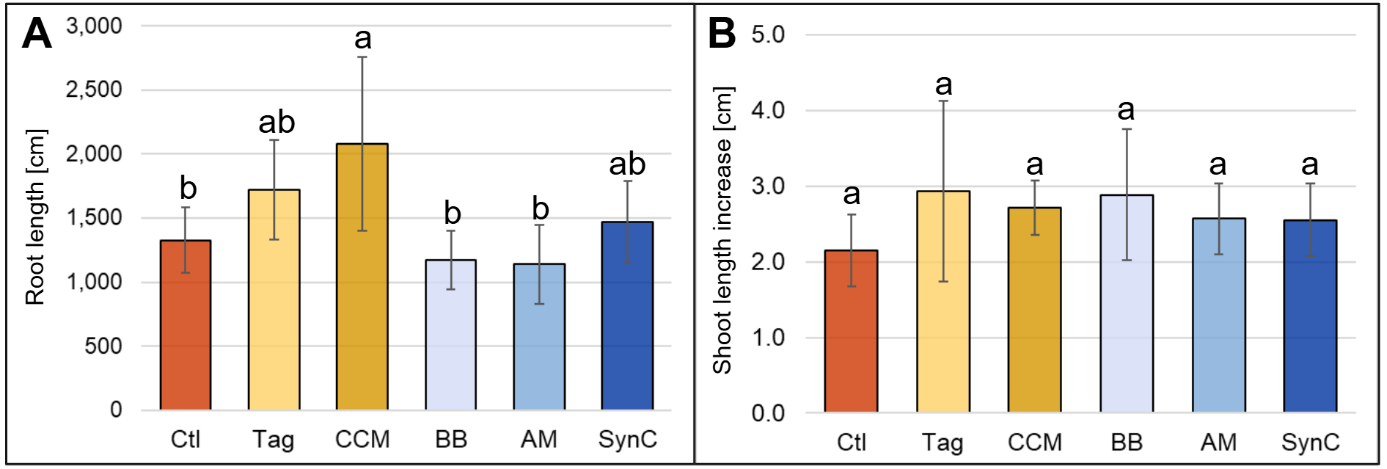


**Fig. S3:** (A) Root length and shoot length increase (B) of apple M.26 after growth in ARD-affected soil for 42 days under different treatments: Ctl: untreated ARD soil; Tag: amendment of *Tagetes patula*; CCM: amendment of a catch crop mixture; BB: inoculation of beneficial bacteria; AM: inoculation of arbuscular mycorrhiza; SynC: inoculation of a synthetic community comprised of BB and AM. Different letters indicate significant differences between treatments according to ANOVA and post-hoc Tukey’s HSD test (*p*<0.05). Given are means and standard deviations of n=6 replicates.


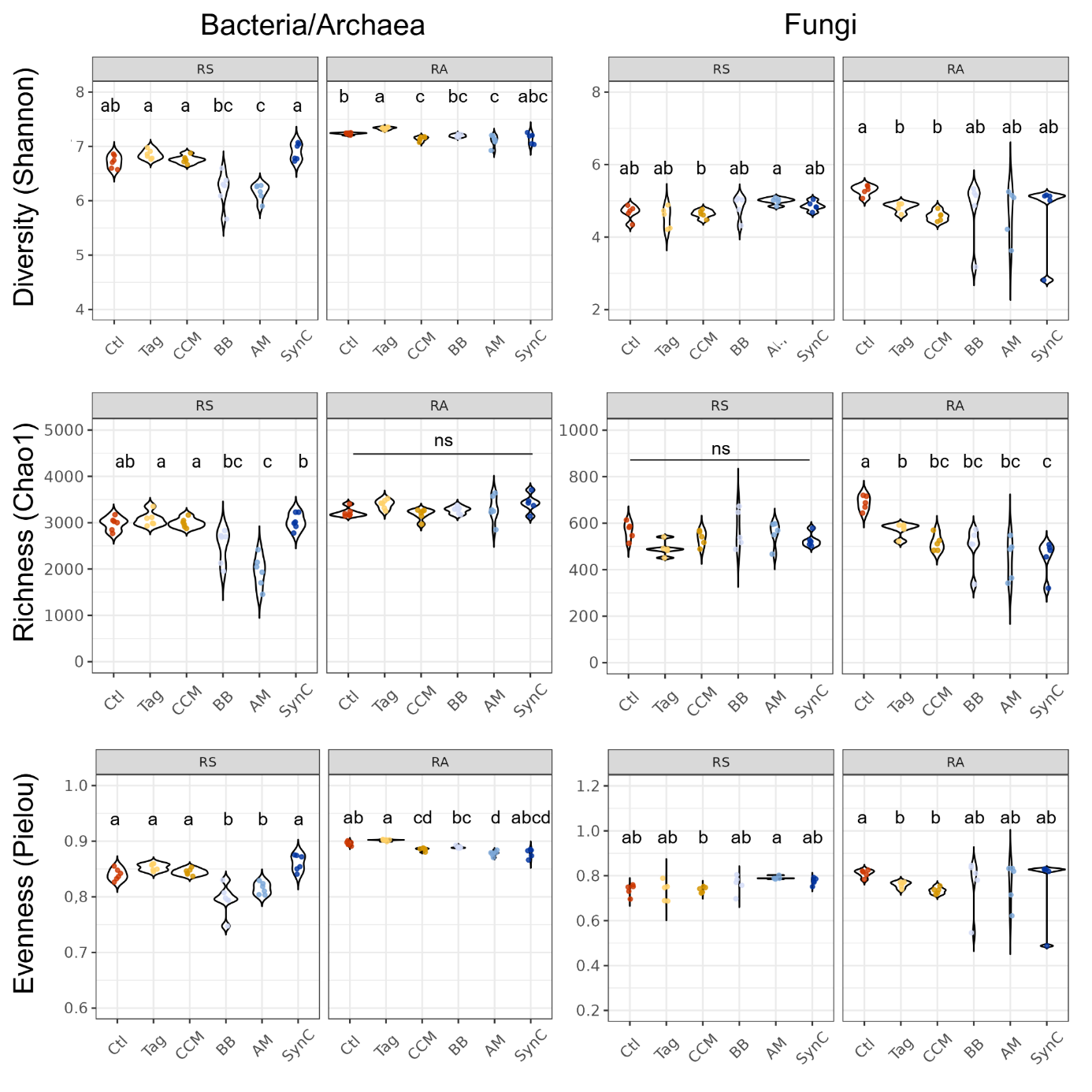


**Fig. S4:** Bacterial/archaeal and fungal alpha-diversity estimates: Shannon diversity, species richness (Chao1), and evenness (Pielou) in the rhizosphere (RS) and root-affected soil (RA) of apple M.26. Plants were grown for seven weeks in untreated ARD soil (Ctl), ARD soil amended with *Tagetes patula* (Tag), a catch crop mixture (CCM), or inoculated with beneficial bacteria (BB), arbuscular mycorrhiza (AM) or a synthetic community comprised of BB and AM (SynC). Different letters indicate significant differences between treatments according to Kruskal-Wallis (*p*<0.05) and paired-Wilcoxon test (*p*-adj< 0.05; Benjamini-Hochberg correction). ns: not significant.

**
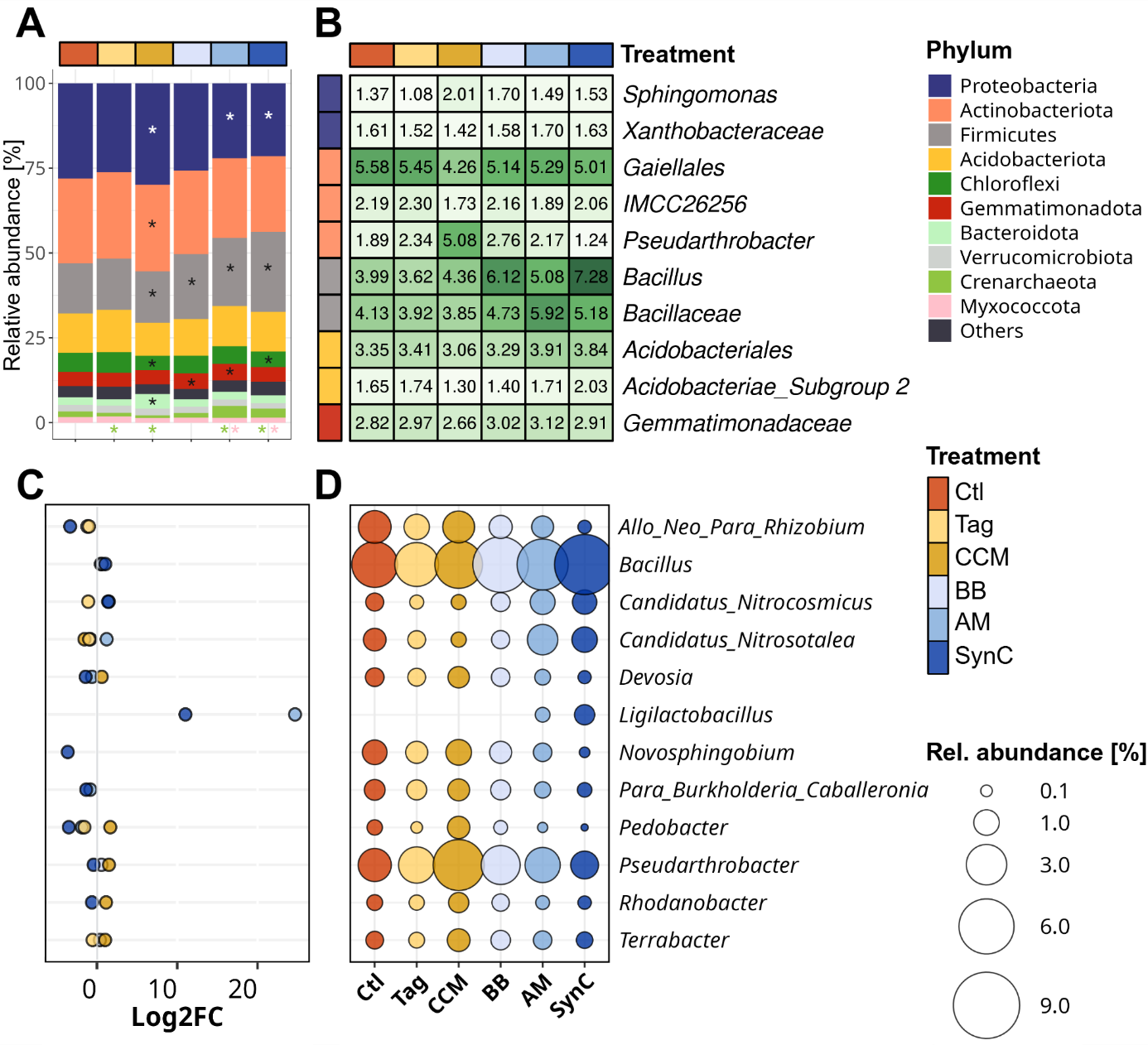
**

**Fig. S5:** Bacterial/archaeal community composition in root-affected soil of M.26 grown for 42 days in ARD soil under different treatments: Ctl: untreated ARD soil; Tag: amendment of *Tagetes patula*; CCM: amendment of a catch crop mixture; BB: inoculation of beneficial bacteria; AM: inoculation of arbuscular mycorrhiza; SynC: inoculation of a synthetic community comprised of BB and AM. Average relative abundance (rel. abundance) of bacterial phyla in the different treatments (A). Asterisks indicate significant differential abundance compared to Ctl. Average rel. abundance of the 10 dominant bacterial taxa across treatments (B). Values in each cell represent average rel. abundances. Log2 fold-change (Log2FC) values (C) and average rel. abundance (D) of differentially abundant taxa between Ctl and treatments Tag, CCM, BB, AM, or SynC. Negative Log2FC values indicate lower abundance, and positive Log2FC values higher abundance compared to Ctl. Dot sizes represent average rel. abundances. Differential abundance testing was done using negative binomial distribution using DeSeq2. Taxa were considered differentially abundant for *p-adj* <0.05 (Benjamini-Hochberg correction). Post-analysis, taxa were filtered for RA>0.5% and Log2FC <-1/>+1. Exact values of relative abundances and Log2FC values are provided in Tab. S3, S5).


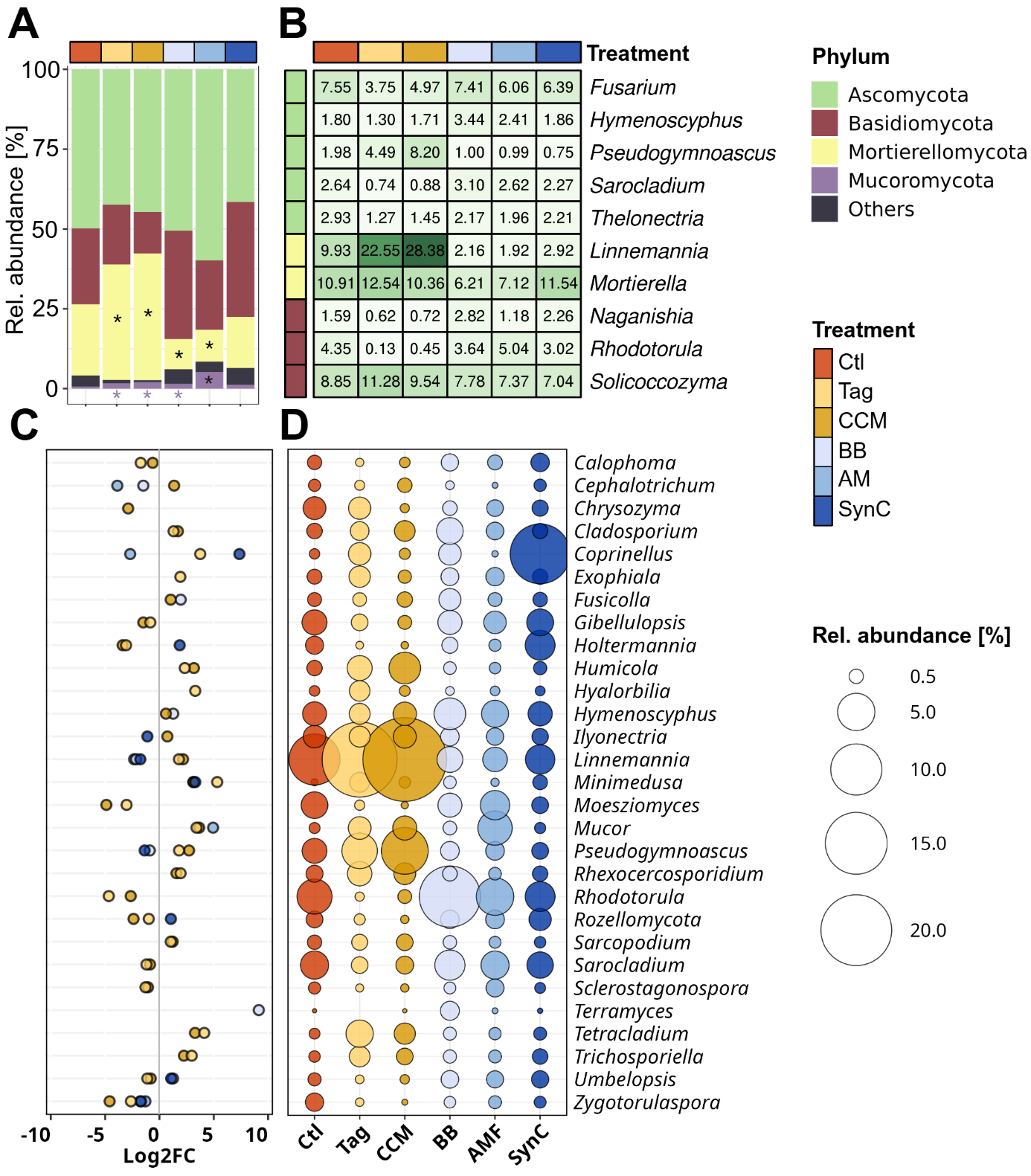


**Fig. S6:** Fungal community composition in root-affected soil of M.26 grown for 42 days in ARD soil under different treatments (n=5): Ctl: untreated ARD soil; Tag: amendment of *Tagetes patula*; CCM: amendment of a catch crop mixture; BB: inoculation of beneficial bacteria; AM: inoculation of arbuscular mycorrhiza; SynC: inoculation of a synthetic community comprised of BB and AM. Average relative abundance (rel. abundance) of bacterial phyla in the different treatments (A). Asterisks indicate significant differential abundance compared to Ctl. Average rel. abundance of the 10 dominant bacterial taxa across treatments (B). Values in each cell represent average rel. abundances. Log2 fold-change (Log2FC) values (C) and average rel. abundance (D) of differentially abundant taxa between Ctl and treatments Tag, CCM, BB, AM, or SynC. Negative Log2FC values indicate lower abundance, and positive Log2FC values higher abundance compared to Ctl. Dot sizes represent average rel. abundances. Differential abundance testing was done using negative binomial distribution using DeSeq2. Taxa were considered differentially abundant for *p-adj* <0.05 (Benjamini-Hochberg correction). Post-analysis, taxa were filtered for RA>0.5% and Log2FC <-1/>+1. Exact values of relative abundances and Log2FC values are provided in Tab. S6, S8).
